# Gastric Cancer Actively Remodels Mechanical Microenvironment to Promote Chemotherapy Resistance via MSCs‐Mediated Mitochondrial Transfer

**DOI:** 10.1002/advs.202404994

**Published:** 2024-10-11

**Authors:** Xin He, Li Zhong, Nan Wang, Baiwei Zhao, Yannan Wang, Xinxiang Wu, Changyu Zheng, Yueheng Ruan, Jianfeng Hou, Yusheng Luo, Yuehan Yin, Yulong He, Andy Peng Xiang, Jiancheng Wang

**Affiliations:** ^1^ Scientific Research Center The Seventh Affiliated Hospital of Sun Yat‐sen University Shenzhen 518107 China; ^2^ Department of Hematology The Seventh Affiliated Hospital of Sun Yat‐sen University Shenzhen 518107 China; ^3^ Digestive Diseases Center Guangdong Provincial Key Laboratory of Digestive Cancer Research The Seventh Affiliated Hospital of Sun Yat‐sen University Shenzhen 518107 China; ^4^ School of Medicine Shenzhen Campus of Sun Yat‐sen University Sun Yat‐sen University Shenzhen 518107 China; ^5^ Shenzhen Key Laboratory of Chinese Medicine Active Substance Screening and Translational Research The Seventh Affiliated Hospital of Sun Yat‐sen University Shenzhen 518107 China; ^6^ State Key Laboratory of Oncology in South China Guangdong Provincial Clinical Research Center for Cancer Sun Yat‐sen University Cancer Center Guangzhou 510060 China; ^7^ Department of Joint and Trauma Surgery The Third Affiliated Hospital of Sun Yat‐sen University Guangzhou 510630 China; ^8^ Center for Stem Cell Biology and Tissue Engineering Key Laboratory for Stem Cells and Tissue Engineering Ministry of Education Sun Yat‐sen University Guangzhou 510080 China; ^9^ National‐Local Joint Engineering Research Center for Stem Cells and Regenerative Medicine Zhongshan School of Medicine Sun Yat‐sen University Guangzhou 510080 China; ^10^ Department of Histoembryology and Cell Biology Zhongshan School of Medicine Sun Yat‐sen University Guangzhou 510080 China

**Keywords:** gastric cancer, matrix stiffness, oxaliplatin resistance, RhoA/ROCK1 signaling pathway

## Abstract

Chemotherapy resistance is the main reason of treatment failure in gastric cancer (GC). However, the mechanism of oxaliplatin (OXA) resistance remains unclear. Here, we demonstrate that extracellular mechanical signaling plays crucial roles in OXA resistance within GC. We selected OXA‐resistant GC patients and analyzed tumor tissues by single‐cell sequencing, and found that the mitochondrial content of GC cells increased in a biosynthesis‐independent manner. Moreover, we found that the increased mitochondria of GC cells were mainly derived from mesenchymal stromal cells (MSCs), which could repair the mitochondrial function and reduce the levels of mitophagy in GC cells, thus leading to OXA resistance. Furthermore, we investigated the underlying mechanism and found that mitochondrial transfer was mediated by mechanical signals of the extracellular matrix (ECM). After OXA administration, GC cells actively secreted ECM in the tumor microenvironment (TEM), increasing matrix stiffness of the tumor tissues, which promoted mitochondria to transfer from MSCs to GC cells via microvesicles (MVs). Meanwhile, inhibiting the mechanical‐related RhoA/ROCK1 pathway could alleviate OXA resistance in GC cells. In summary, these results indicate that matrix stiffness could be used as an indicator to identify chemotherapy resistance, and targeting mechanical‐related pathway could effectively alleviate OXA resistance and improve therapeutic efficacy.

## Graphical abstract

Extracellular mechanical signaling plays a crucial role in the OXA resistance in gastric cancer. After OXA administration, GC cells actively secrete ECM to increase matrix stiffness and promote mitochondria to transfer from MSCs to GC cells via microvesicles, which reduces the levels of mitophagy, resulting in OXA resistance. Targeting mechanical‐related pathway could alleviate OXA resistance and improve OXA therapeutic efficacy.

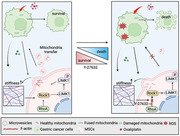

## 1 Introduction

Gastric cancer is one of the most common malignant tumors of the digestive tract. Over 1 million new cases of gastric cancer were estimated globally in 2020, resulting in 768,793 deaths.^[^ ^1^ ^]^ At present, the treatment of gastric cancer mainly relies on surgery, but it would be combined with multidisciplinary comprehensive treatment models such as chemotherapy. Approximately 50% of patients experience recurrence or metastasis within two years after surgery, with a low 5‐year survival rate, posing a serious threat to human life and health.^[^ ^2^ ^]^

Oxaliplatin as a third‐generation platinum‐based anti‐tumor drug, has shown good effects on tumors; Compared to cisplatin, it greatly reduces nephrotoxicity and ototoxicity.^[^ ^3^ ^]^ However, chemotherapy resistance is the main cause of treatment failure in gastric cancer. Current researches on the OXA resistance mechanisms mainly focus on the tumor itself. First, DNA repair mechanisms of tumor cells. Tumor cells reduce OXA‐induced DNA damage by enhancing the DNA repair mechanism, thereby reducing the efficacy of OXA.^[^ ^4^ ^]^ Second, alterations in drug transport channels within tumors. Abnormal channels lower the concentration of OXA within the tumor and reduce the efficacy of OXA.^[^ ^5^ ^]^ Third, impairments in the apoptosis pathways of tumor cells. Mutations or defects in genes related to the cell apoptosis pathway may occur in tumor cells, leading to blocked apoptosis and a diminished therapeutic effect of OXA.^[^ ^6^ ^]^ In this paper, we mainly focus on the tumor microenvironment (TME) and deeply explore the mechanisms of the microenvironment involvement in OXA resistance, which plays a very important role in the treatment and prognosis of gastric cancer.

In recent years, TME has played an important role in drug resistance.^[^ ^7^ ^]^ TME consists of both non‐cellular components such as the extracellular matrix, and a wide variety of cellular components, including MSCs, MSCs‐derived fibroblasts known as cancer‐associated fibroblasts and endothelial cells, and so on.^[^ ^7c^ ^]^ The role of TME in drug resistance has become a hot research field. For example, some secreted proteins produced by MSCs, such as IL‐8,^[^ ^8^ ^]^ IL‐1β,^[^ ^9^ ^]^ IL‐6,^[^ ^10^ ^]^ IGF‐1, IGF‐2,^[^ ^11^ ^]^ and TGF‐β,^[^ ^12^ ^]^ are associated with chemotherapy resistance. On the other hand, exosomes, such as miR‐98‐5p,^[^ ^13^ ^]^ also participate in chemotherapy resistance. However, most of the researches mainly focus on cell line experiments in vitro and in vivo, and the mechanism analysis based on clinical samples is lacking.

In this article, we first analyzed OXA‐resistant gastric cancer samples using single‐cell sequencing and found that the mitochondrial content of GC cells increased in a biosynthesis‐independent manner. In vitro and in vivo experiments showed that the increased mitochondria of GC cells were mainly derived from MSCs. Further, we found that mitochondrial transfer was mediated by extracellular matrix stiffness. After OXA administration, GC cells actively remodelled TME by increasing ECM accumulation. Mechanically, high matrix stiffness activated the RhoA/ROCK1 signaling pathway of MSCs and promoted more mitochondria to transfer to GC cells via MVs. Meanwhile, we found that the tumor‐killing effect of OXA was restored after blocking mechanical‐related signaling pathways. These data suggest that mechanical signaling plays an important role in OXA resistance in gastric cancer.

## 2 Results

### 2.1 Gastric Cancer Cells Increase Mitochondrial Content in Biosynthesis‐Independent Manner after OXA Resistance

**Figure 1:**
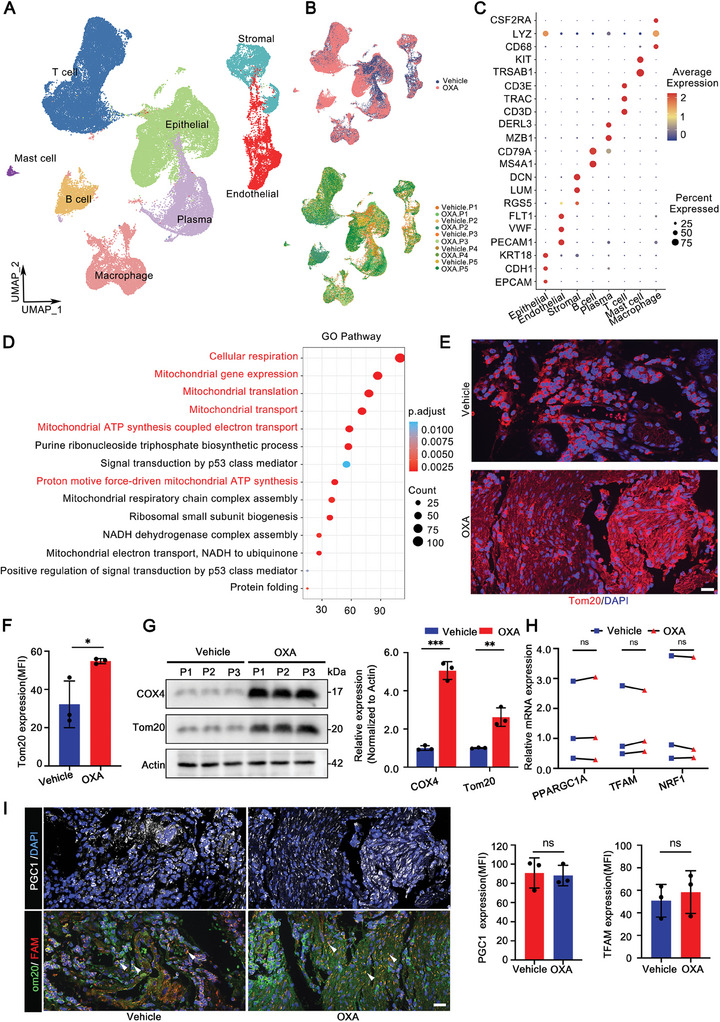
Mitochondrial content increased in OXA‐resistant GC cells. A) UMAP plot of cell clusters in gastric cancer tissues. B) UMAP plot showed cell origin by color, Vehicle or OXA origin (upper panel), and patient origin (bottom panel). C) The bubble plot showed the expression of distinctive marker genes in eight cell types. The dot size indicates the percentage of gene expression, and the dot color indicates the average expression level. D) The bubble plot showed that differential expressed genes in the Vehicle group and OXA group were significantly enriched in the gene sets related to mitochondria. E) Representative immunofluorescence images displayed the mitochondrial content (Tom20) in gastric cancer tissues. Scale bar: 20 µm. F) MFI quantification of Tom20 in (E) (*n* = 3). G) Western Blot analysis and quantification of COX4 and Tom20 in Vehicle group and OXA group (*n* = 3). H) qRT‐PCR analysis of PPARGC1A, TFAM, and NRF1 mRNA levels in Vehicle group and OXA group (*n* = 3). I) Representative immunofluorescence images and MFI quantification of PGC‐1α and TFAM (*n* = 3). Scale bar: 20 µm. The data above are presented as mean ± S.D. of three independent experiments, *P*‐values are calculated between two groups was performed using an unpaired *t*‐test, and multiple‐group statistical analysis was performed using one‐way analysis of variance (anova) followed by the Tukey multiple‐comparison test. ns, not significant; *P < 0.05; **P < 0.01; ***p < 0.001.

In order to explore the underlying mechanisms of OXA resistance, single‐cell RNA sequencing (scRNA‐seq) was performed on the GC samples from the same patient before and after OXA administration. We first performed quality control to ensure data quality was sufficient for downstream analysis. After cell quality control (Figure S1A,B, Supporting Information), dimensionality reduction and unsupervised cell clustering, we identified Epithelial (EPCAM, CDH1, KRT18), Endothelial (PECAM1, VWF, FLT1), Stromal (RGS5, LUM, DCN), B cell (CD79A, MS4A1), Plasma (MZB1, DERL3), T cell (CD3D, TRAC, CD3E), Mast cell (TRSAB1, KIT) and Macrophage (CD68, LYZ, CSF2RA) as eight distinct lineages based on the expression of marker genes (**Figure** **1**A–C).^[^ ^14^ ^]^ In order to distinguish GC cells from normal epithelial cells in Epithelial cell clustering, interCNV analysis has been performed (Figure S1C,D, Supporting Information). This analysis indentified 12,305 epithelial cells as GC cells, and other 5,649 epithelial cells were identified as normal epithelial cells. We excluded normal epithelial cells in the downstream analysis. Next, we further conducted Gene ontology (GO) enrichment analysis, the results showed that mitochondrial function‐related biology features were enriched, such as cellular respiration, mitochondrial gene expression, mitochondrial transport, and mitochondrial ATP synthesis coupled electron transport, suggesting that mitochondria played an important role in OXA resistance in GC cells (Figure 1D). Previous researches reported that mitochondrial content is related to the mitochondrial function.^[^ ^15^ ^]^ Therefore, we first investigated the mitochondrial content of the Vehicle group and OXA group by immunofluorescence. The results showed the expression of outer membrane protein (Tom20), a mitochondrial marker, increased after OXA resistance (Figure 1E,F). Meanwhile, we also found increased protein levels of mitochondrial inner membrane protein (COX4) and Tom20by Western Blot (Figure 1G). These findings suggest that OXA resistance is associated with an increase in the mitochondrial content within GC cells. The mitochondrial content of GC cells depends on endogenous mitochondrial biosynthesis and exogenous mitochondrial transfer.^[^ ^16^ ^]^ Thus, we examined the expression levels of genes related to mitochondrial biosynthesis by qRT‐PCR. The results showed that there was no significant difference in the levels of mitochondrial biosynthesis‐related genes between the Vehicle and OXA group (Figure 1H). Further, we also found that protein levels of PGC1α (a key regulator of mitochondrial biosynthesis) and TFAM (a mitochondrial nucleoid marker) were not significantly increased in the OXA‐resistant group (Figure 1I). Taken together, these results indicate that GC cells increase mitochondrial content in a biosynthesis‐independent manner after OXA resistance.

### 2.2 OXA Treatment Induces Mitochondria Transfer from MSCs to GC Cells

**Figure 2:**
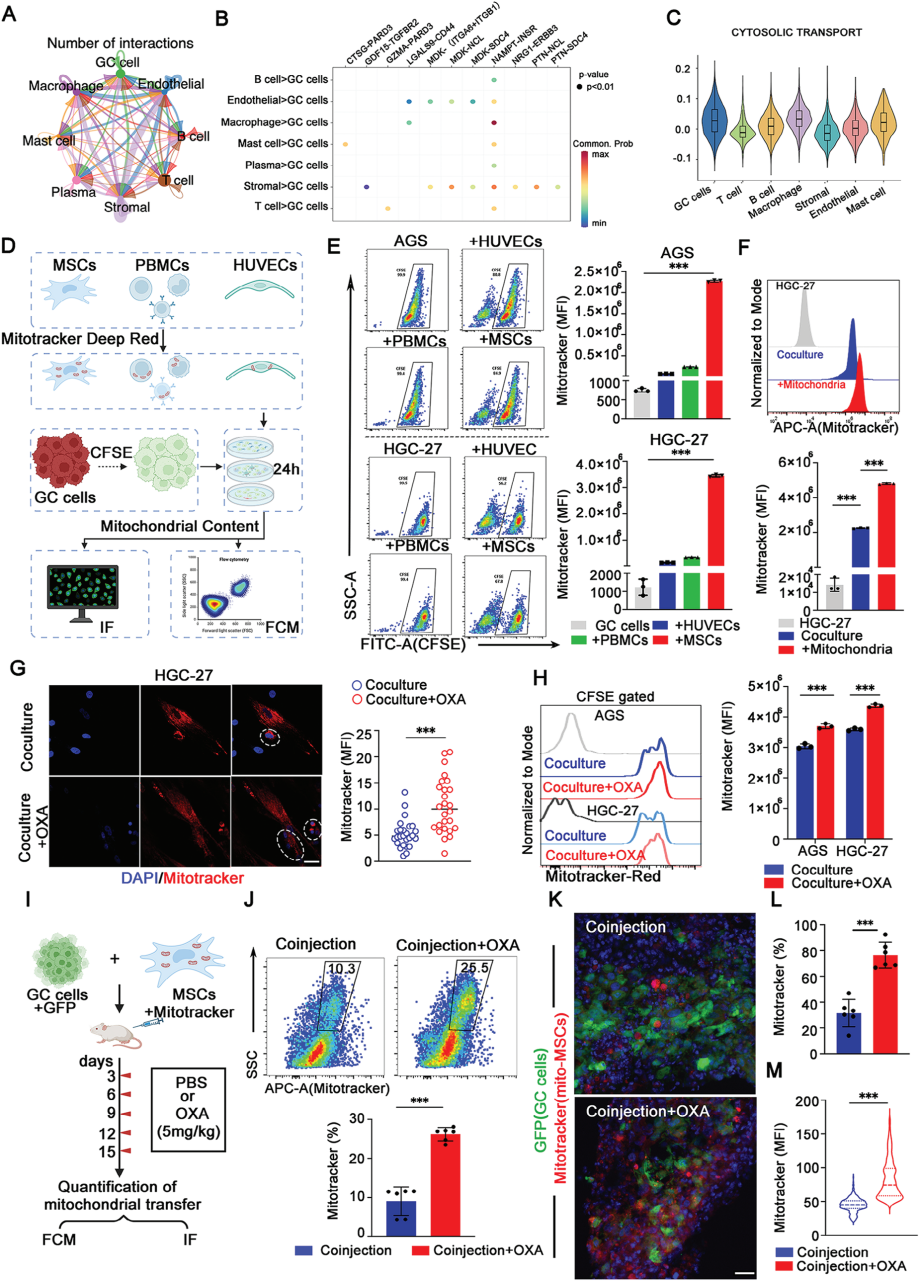
OXA treatment induces mitochondria transfer from MSCs to GC cells. A) The net plot showed the number of interactions between various cell types. Thicker lines represent more interactions. B) Receptor‐ligand pairs in cancer cells and other cell types. The dot size represents the *P* value, and the color represents the communication probability. C) The enrichment scores of the cytosolic transport gene set among different cell types. D) Schematic overview of Mitotracker Deep Red‐labeled MSCs or PBMCs or HUVECs cocultured with CFSE‐labeled GC cells system followed by flow cytometry and immunofluorescence. E) Flow cytometry analysis of mitochondrial content in GC cells after coculture for 24 h. The quantification of the MFI of each group was analyzed and graphed (*n* = 3). F) Flow cytometry analysis of Mitotracker in each group (HGC‐27, HGC‐27‐MSCs, and HGC‐27+Mitochondria). The quantification of the MFI of each group was analyzed and graphed (*n* = 3). G) Representative immunostaining images of mitochondrial transfer in each group (HGC‐27‐MSCs and HGC‐27‐MSCs+OXA). The cells in the white circle indicates that HGC‐27 contained MSCs‐derived mitochondria. Quantification of MFI of MitoTracker Deep Red uptaken by HGC‐27 shown on the right (*n* = 3). Scale bar: 30 µm. H) Flow cytometry analysis of Mitotracker Red in each group (MSCs‐GC cells, and MSCs‐GC cells+OXA). The quantification of the MFI of each group was analyzed and graphed (*n* = 3). I) Schematic diagram of a xenograft model. GFP‐labeled HGC‐27 cells were mixed with MitoTracker Deep Red‐labeled MSCs, subcutaneously coinjected into the left flank of immunocompetent mice, HGC‐27‐bearing mice received injections of OXA (5 mg kg^−1^) or PBS, 15 days later, mice were sacrificed, and mitochondrial transfer was analyzed and quantified (*n* = 6 mice group^−1^). J) Flow cytometry analysis of MitoTracker Deep Red in each group (MSCs‐GC cells coinjection, and MSCs‐GC cells coinjection+OXA). Quantification of MitoTracker Deep Red of each group was analyzed and graphed (*n* = 6 mice group^−1^). K) Representative immunostaining images of mitochondrial transfer in each group in vivo (MSCs‐GC cells coinjection, and MSCs‐GC cells coinjection+OXA). Scale bar: 30 µm. L) Quantification of MitoTracker Deep Red of each group was analyzed and graphed. The analysis was presented as a percentage of mitochondrial transfer (*n* = 6 mice group^−1^). M) Quantification of MFI of MitoTracker Deep Red uptaken by GC cells (*n* = 6 mice group^−1^). The data above are presented as mean ± S.D. of three independent experiments. *P*‐values are calculated between two groups was performed using an unpaired *t*‐test, and multiple‐group statistical analysis was performed using one‐way analysis of variance (anova) followed by the Tukey multiple‐comparison test. ns, not significant; *P < 0.05; **P < 0.01; ***P < 0.001.

According to the above results, we found that the increased mitochondrial content in the OXA‐resistant group is not endogenous synthesis. Thus, we supposed that these increased mitochondria might originate from the tumor microenvironment. Then, we analyzed the scRNA‐seq data and found that there were numerous cell‐cell interactions in the tumor microenvironment (**Figure** **2**A,B; Figure S2A, Supporting Information). Among them, stromal cells, immune cells, and endothelial cells played a crucial role in the cytosolic transport of OXA‐resistant GC cells (Figure 2C). To further investigate the origin of the increased mitochondria within the tumors, we selected MSCs, peripheral blood mononuclear cells (PBMCs), and human umbilical vein endothelial cells (HUVECs) to coculture with GC cells. MSCs, PBMCs, and HUVECs were labeled with MitoTracker Deep Red, and GC cells were labeled with CFSE. Prior to coculture, we first normalized MSCs, PBMCs, and HUVECs according to the mean fluorescence intensity (MFI) of MitoTracker to ensure equal numbers of the three cell types. Then we quantified mitochondrial transfer by flow cytometry and immunofluorescence after 24h with coculture system (Figure 2D). The results showed a significant increase of mitochondrial content in the MSCs‐GC cells coculture system (Figure 2E; Figure S2B,C, Supporting Information), indicating that MSCs played a central role in transferring mitochondria to GC cells. Furthermore, we isolated mitochondria from MSCs and verified their function by confocal microscopy and ATP assays (Figure S2D, Supporting Information). After MSCs‐derived mitochondria were cocultured with GC cells, we found that GC cells could take up mitochondria exponentially (Figure 2F; Figure S2E, Supporting Information).

To explore whether OXA treatment promotes mitochondria to transfer from surrounding cells to GC cells, we treated coculture systems with OXA. The results showed that the mitochondria of MSCs were transferred significantly after OXA treatment in the coculture system (Figure 2G,H; Figure S3SA–C, Supporting Information). Furthermore, we co‐injected GFP‐labeled GC cells and MitoTracker Deep Red‐labeled MSCs subcutaneously into immunodeficient mice. These tumor‐bearing mice were then injected with either OXA (5 mg kg^−1^) or PBS every 3 days since day 3, and tumors were harvested at the 15th day for immunofluorescence staining and flow cytometry (Figure 2I). The results showed that the uptake of MSCs‐derived mitochondria by GC cells increased significantly after OXA treatment (Figure 2J). Analogously, the MFI of mitochondria in GC cells also increased after OXA treatment, suggesting that OXA treatment promoted mitochondria to transfer from MSCs to GC cells in vivo (Figure 2K–M). It has been reported that the direction of mitochondrial transfer between cells is bidirectional,^[^ ^17^ ^]^ so we measured whether  mitochondria could transfer from GC cells to MSCs. The results showed that the number of mitochondria in MSCs did not increase significantly after OXA treatment (Figure S3D, Supporting Information). To sum up, our results demonstrate that OXA treatment promotes the mitochondria transfer from MSCs to GC cells, and the direction of mitochondrial transfer is unidirectional, from MSCs to GC cells.

### 2.3 MSCs Transfer Mitochondria to GC Cells via Microvesicles (MVs)

**Figure 3:**
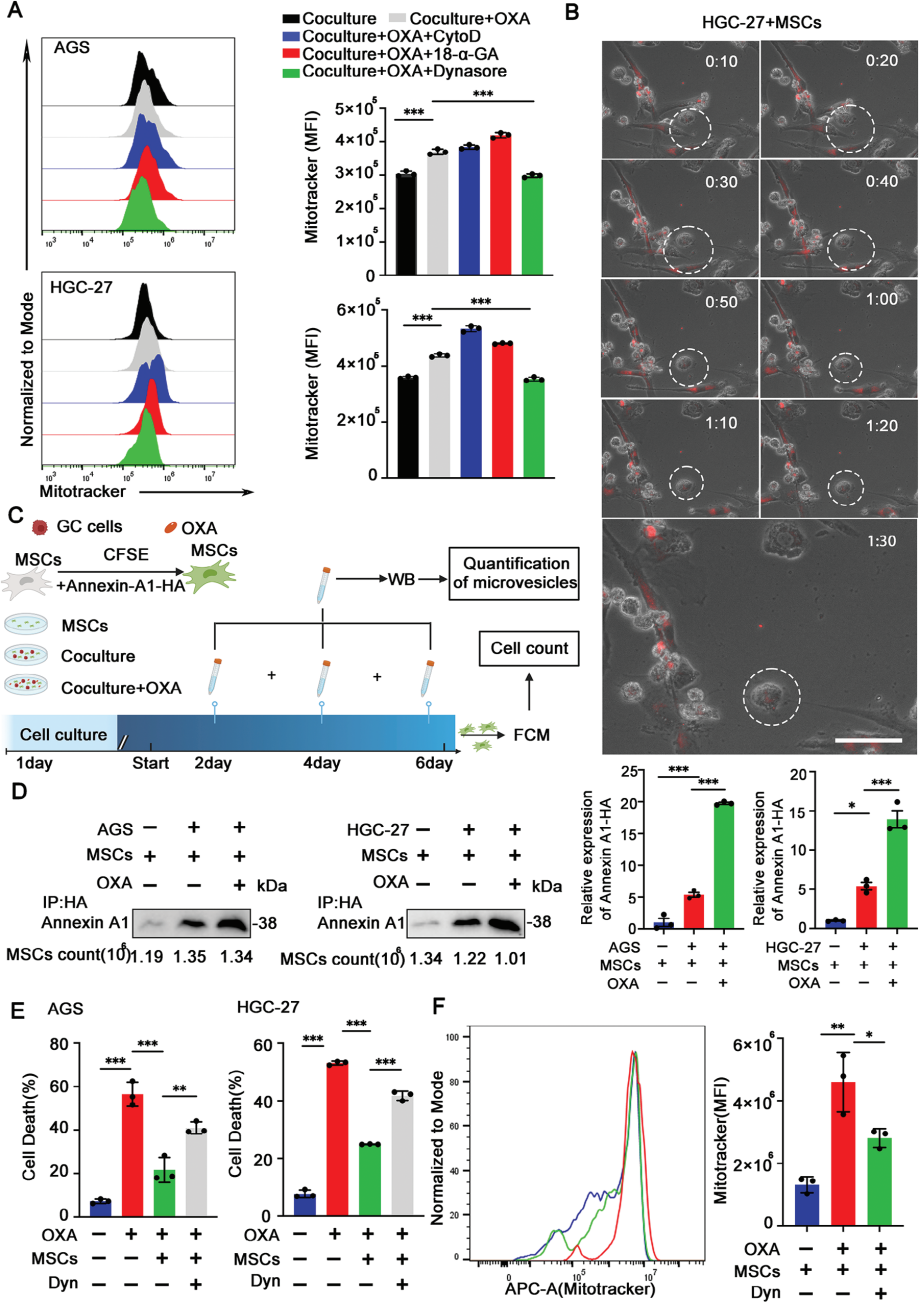
MSCs transfer mitochondria to GC cells via MVs. A) Flow cytometry analysis of MitoTracker Red uptaken by GC cells (CFSE+ gated). CFSE‐labeled GC cells were cocultured with MitoTracker Red‐labeled MSCs, treated with OXA (5 mg kg^−1^), and added with 18‐α‐GA (50 µm), dynasore (50 µm) or cytochalasin D (200 nm) inhibitors (*n* = 3). B) Representative living cell tracing images of mitochondrial transfer in HGC‐27‐MSCs coculture system after OXA treatment for 24 h (*n* = 3). MSCs were labeled with MitoTracker Deep Red before coculture with HGC‐27 cells. Scale bar: 50 µm. C) Schematic diagram of MSCs‐GC cells coculture system. MSCs were labeled with CFSE staining and transfected with lentivirus consisting of HA‐tag Annexin‐A1. The media of the MSCs‐GC cells coculture system was collected at day 2, 4, and 6 after OXA treatment, and the protein levels of MVs in the media was detected by co‐immunoprecipitation. The number of CFSE‐positive MSCs was counted by flow cytometry. D) Co‐immunoprecipitation analysis of Annexin‐A1 in each group (MSCs, MSCs‐GC cells, and MSCs‐GC cells+OXA). Quantitative analysis of the Annein‐A1 levels shown on the right (*n* = 3). E) The mortality rate of GC cells after 24 h treatment in each group was analyzed by calcein‐AM/PI staining (*n* = 3). F) Flow cytometry analysis of MitoTracker Red uptaken by GC cells in each group in vivo (*n* = 3 mice group^−1^). The data above are presented as mean ± S.D. of three independent experiments. *P*‐values are calculated between two groups was performed using an unpaired *t*‐test, and multiple‐group statistical analysis was performed using one‐way analysis of variance (anova) followed by the Tukey multiple‐comparison test. ns, not significant; *P < 0.05; **P < 0.01; ***P < 0.001.

Mitochondrial transfer between cells has been reported to be mediated by tunneling nanotubes (TNTs), MVs, and gap junctions.^[^ ^18^ ^]^ Among them, TNT is the main manner of mitochondria transfer.^[^ ^19^ ^]^ Thus, we initially detected whether the TNT inhibitor (Cytochalasin D, Cyto D) could block the mitochondrial transfer in MSCs‐GC cells coculture system after OXA treatment by immunofluorescence. The results showed that Cyto D had no effect on the mitochondria transfer from MSCs to GC cells after OXA treatment (Figure S4A,B, Supporting Information). We then treated the MSCs‐GC cells coculture system with other inhibitors, including 18‐α‐GA (an inhibitor of gap junctions), dynasore (an inhibitor of MVs), and measured the mitochondrial transfer by flow cytometry. The results showed that only dynasore blocked the effect of OXA on mitochondrial transfer (**Figure** **3**A), suggesting that MVs were the main manner of mitochondria transfer from MSCs to GC cells after OXA treatment. To more directly observe mitochondrial transfer, we used live‐cell imaging to observe the dynamics of live cells at a time interval of 0 to 90 min. After 90 min, MSCs‐derived mitochondria (indicated in red) enter GC cells (highlighted by white line area) via MVs (Figure 3B; Figure S4C, Supporting Information). It is well known that MVs play a key role on material communication among cells.^[^ ^20^ ^]^ Therefore, we further investigated the effect of OXA treatment on the secretion of MVs by MSCs. We transfected the MSCs with lentivirus encoding HA‐tagged Annexin‐A1 (a marker for MVs), collected supernatant, and detected MSCs‐derived MVs by co‐immunoprecipitation with HA‐tagged antibody (Figure 3C). The results showed that OXA treatment significantly promoted MSCs to secrete MVs in MSCs‐GC cells coculture system (Figure 3D). Considering that mitochondria played a vital role in cell survival,^[^ ^19^ ^]^ we speculated that mitochondrial transfer promoted the survival of GC cells. Thus, we performed a cell viability assay, and the results showed that MSCs weakened the tumor‐killing effect of OXA, but this effect could be inhibited by dynasore (Figure 3E; Figure S4D,E, Supporting Information). Additionally, we further investigated the mitochondria transfer in vivo (Figure S4F, Supporting Information). The results showed that OXA treatment promoted the MSCs to transfer mitochondria to GC cells, while dynasore inhibited this process (Figure 3F). In summary, these results indicate that OXA treatment promotes the mitochondria to transfer from MSCs to GC cells via MVs, thereby improving the survival of GC cells.

### 2.4 MSCs‐Derived Mitochondria Restore GC Cells Mitochondrial Function by Reducing Mitophagy Levels

**Figure 4:**
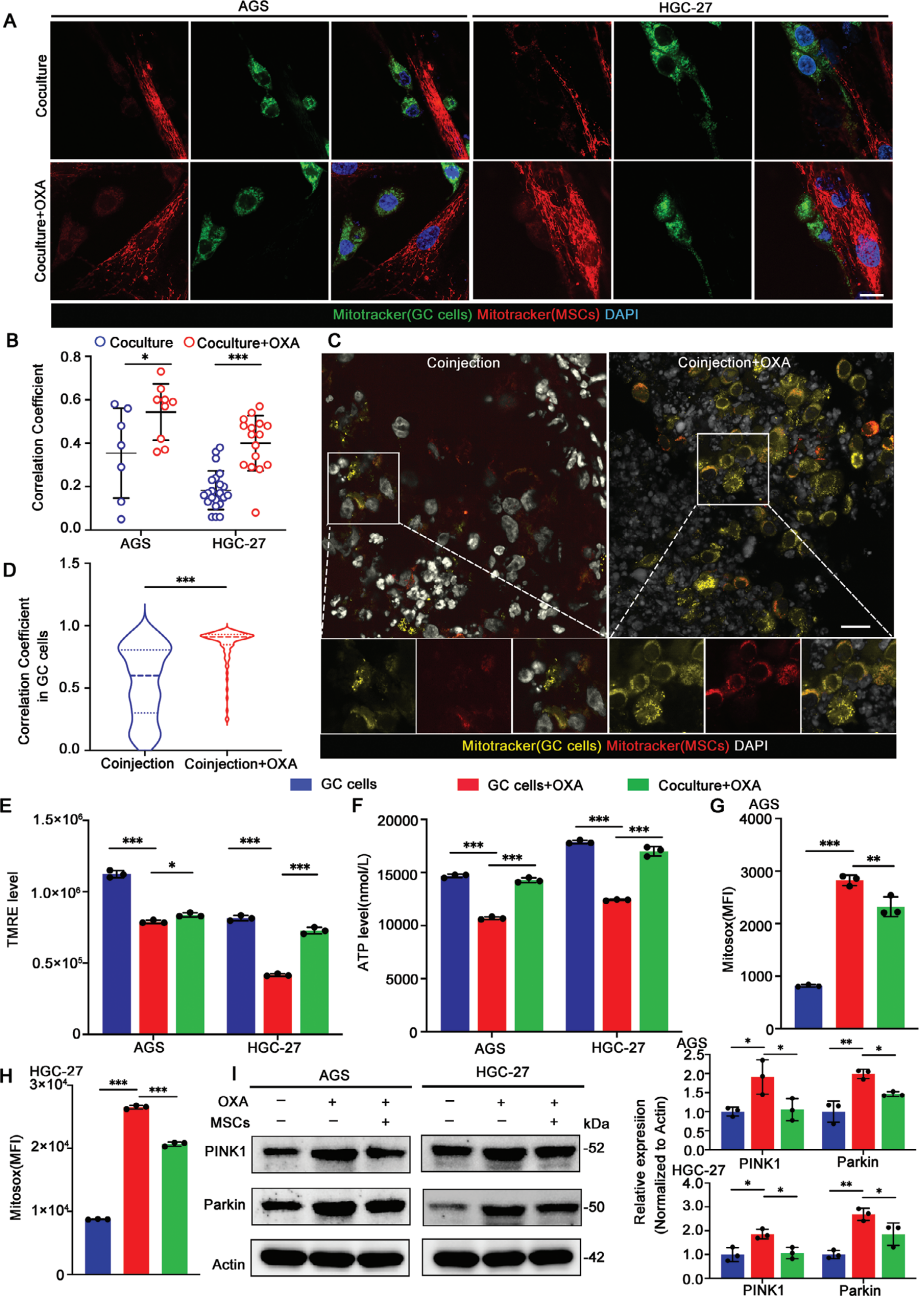
MSCs‐derived mitochondria restore GC cells mitochondrial function by reducing mitophagy levels. A) Representative immunostaining images of fused mitochondria in each group (MSCs‐GC cells and MSCs‐GC cells+OXA). MitoTracker Red‐labeled MSCs and MitoTracker Deep Red‐labeled GCs. Scale bar: 10 µm. B) Quantification of the levels of colocalization in (A) (*n* = 4). C) Representative immunofluorescence images of mitochondrial fusion in each group in vivo (MSCs‐GC cells or MSCs‐GC cells+OXA). Scale bar: 30 µm. D) Quantification of the levels of colocalization in (C) (*n* = 3). E) Quantitative analysis of the TMRE in each group (GC cells, GC cells+OXA, and MSCs‐GC cells+OXA) (*n* = 3). F) Measurement of the intracellular ATP levels in each group (GC cells, GC cells+OXA, and MSCs‐GC cells+OXA) (*n* = 3). G,H) ROS levels in each group (GC cells, GC cells+OXA, and MSCs‐GC cells+OXA) were analyzed by flow cytometry. Mitochondrial ROS was probed with Mitosox (PE channel) (*n* = 3). I) Western Blot analysis of mitophagy‐regulated proteins (PINK1 and Parkin) in each group (GC cells, GC cells+OXA, and MSCs‐GC cells+OXA). Quantitative analysis shown on the right (*n *= 3). The data above are presented as mean ± S.D. of three independent experiments. *P*‐values are calculated between two groups was performed using an unpaired *t*‐test, and multiple‐group statistical analysis was performed using one‐way analysis of variance (anova) followed by the Tukey multiple‐comparison test. ns, not significant; *P < 0.05; **P < 0.01; ***P < 0.001.

The results had proved that OXA treatment promoted the mitochondria transfer from MSCs to GC cells, but the role of MSCs‐derived mitochondria was unclear. According to the literatures,^[^ ^21^ ^]^ we first examined the co‐localization of the mitochondria of GC cells (indicated in green) and the mitochondria of MSCs (indicated in red) to assess the levels of mitochondrial fusion. The results showed that GC cells‐derived mitochondria and MSCs‐derived mitochondria were high co‐localized after OXA treatment (**Figure** **4**A,B; Figure S5A, Supporting Information), suggesting that OXA promoted mitochondrial fusion. Further, we co‐injected MitoTracker Red‐labeled GC cells and MitoTracker Deep Red‐labeled MSCs into immunodeficient mice. After a 15‐day implantation period, the subcutaneous tumors were harvested to perform immunofluorescence. Similarly, the results also showed that OXA treatment promoted mitochondrial fusion in GC cells in vivo (Figure 4C,D). To further explore the function of mitochondria, we examined the levels of mitochondrial membrane potential, ATP production and mitochondrial ROS. The results showed that the function of mitochondria in GC cells reduced after OXA treatment, while restored in the MSCs‐GC cells coculture system (Figure 4E–H). These findings indicated that the MSCs‐derived mitochondria restored the function of mitochondria in GC cells. Additionally, excessive mitophagy is reported to a potential mechanism of OXA‐induced tumor cell death.^[^ ^22^ ^]^ However, we found that in the MSCs‐GC cells coculture system, the levels of mitophagy of GC cells decreased (Figure 4I; Figure S5B–E, Supporting Information). To further prove that the role of mitophagy in OXA resistance, we constructed the over‐expressing PINK1 GC cells and determined the cell viability by calcein‐AM/PI staining. The results showed that after OXA treatment, MSCs promoted the OXA resistance of GC cells and increased cell survival, while overexpress PINK1 significantly weakened this process (Figure S5F, Supporting Information). Together, these results indicate that OXA treatment promotes the mitochondrial transfer of MSCs to GC cells, contributes to the recovery of mitochondrial function of tumor cells, reduces the levels of mitophagy of GC cells, and ultimately enables GC cells to survive.

### 2.5 OXA‐Resistant GC Cells Increase Extracellular Matrix Stiffness to Actively Reshape the Tumor Microenvironment

**Figure 5:**
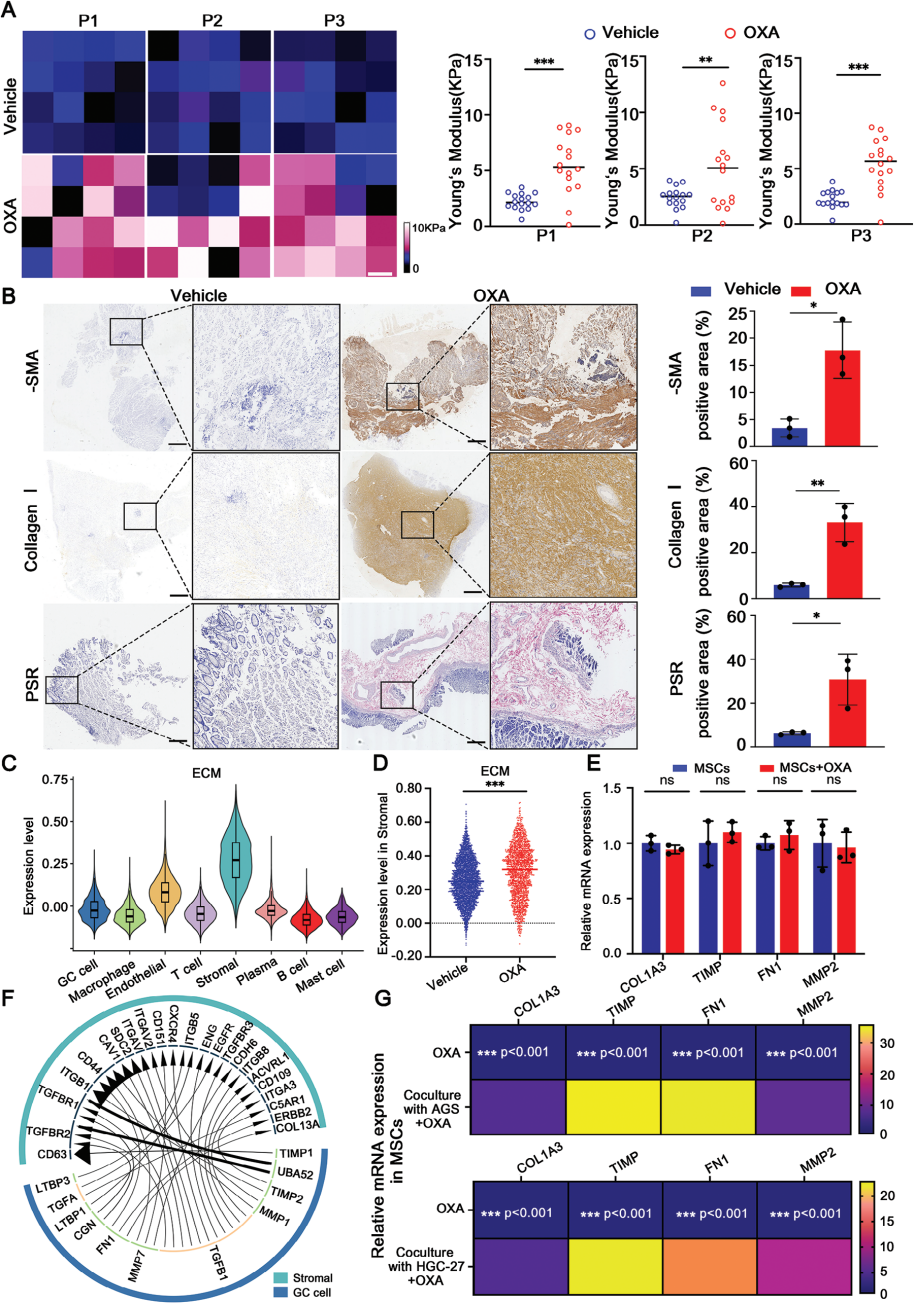
OXA resistance increases the matrix stiffness. A) Atomic force images and statistical analysis in tissues (*n* = 3). Scale bar: 700 µm. B) Representative IHC images of GC tissues and quantitative analysis of the levels of α‐SMA, collagen I, and PSR (*n* = 3). The images were obtained with a 4× magnification lens. Scale bar: 700 µm. C) The evaluated activity scores of the ECM in GC cell, Macrophage, Endothelial, T cell, Stromal, Plasma, B cell, and Mast cell after OXA treatment. D) The evaluated activity scores of the ECM in stromal cells in the Vehicle group and OXA group. E) qRT‐PCR analysis of COL1A3, TIMP, FN1 and MMP2  mRNA levels in MSCs in each group (MSCs and MSCs+OXA) (*n* = 3). F) The interactions between GC cells and stromal cells were enriched in ECM ligands and receptors. Thick means strong, thin means weak. G) qRT‐PCR analysis of COL1A3, TIMP, FN1 and MMP2  mRNA levels in MSCs in each group (MSCs+OXA and MSCs‐GC cells+OXA) (*n* = 3). The data above are presented as mean ± S.D. of three independent experiments. *P*‐values are calculated between two groups was performed using an unpaired *t*‐test, and multiple‐group statistical analysis was performed using one‐way analysis of variance (anova) followed by the Tukey multiple‐comparison test. ns, not significant; *P < 0.05; **P < 0.01; ***P < 0.001.

The secretion of the MVs depends on the cytoskeleton, which senses extracellular mechanical signals from the extracellular environment.^[^ ^23^ ^]^ Therefore, we first compared the elastic modulus of the Vehicle and OXA group. The results showed that the elastic modulus of the OXA group was significantly increased (**Figure** **5**A). Besides, the stiffness of the matrix was reported to be related to tissue fibrosis and extracellular matrix deposition.^[^ ^24^ ^]^ Thus, we next measured the protein levels of α‐SMA and collagen I, and the deposition levels of collagen (detected by PSR staining). The results showed that α‐SMA and collagen I levels, as well as collagen deposition levels, significantly increased in the OXA group (Figure 5B), suggesting that OXA resistance promoted the accumulation of extracellular matrix and enhanced the matrix stiffness. For further exploring the mechanism of matrix stiffness increase after OXA resistance, we analyzed the scRNA‐seq data and found that the ECM score of stromal cells was the highest, and after OXA resistance, the levels of ECM‐related RNA expression also increased significantly (Figure 5C,D), suggesting that stromal cells played a vital role in ECM deposition after OXA resistance. Next, we treated the monoculture MSCs with OXA and extracted the mRNA of MSCs. Unexpectedly, qRT‐PCR results showed that there were no significant changes in the expression of ECM‐related genes between the MSCs group and MSCs+OXA group (Figure 5E), suggesting that OXA‐induced ECM secretion by MSCs required the involvement of other cells in the tumor microenvironment. Previous researches reported that cancer cells promote MSCs to secret various proteins such as collagen, cytokines, and chemokines.^[^ ^25^ ^]^ Thus, we analyzed scRNA‐seq data and found that GC cells regulated stromal cells to secret ECM through enhanced ligand‐receptor binding (Figure 5F). Further, we found that after OXA treatment, the expression of ECM‐related genes in MSCs increased with the MSCs‐GC cells coculture systems (Figure 5G), indicating that GC cells played an important role in promoting the expression of ECM‐related genes in MSCs. Interestingly, we also found the OXA promoted mRNA levels of ECM components in GC cells (Figure S6A, Supporting Information). These results suggest that the ECM is mainly secreted by MSCs and GC cells. To further confirm the conclusions that OXA treatment promoted the ECM accumulation and enhanced the ECM stiffness, we co‐injected subcutaneously the MSCs‐GC cells into immunodeficient mice. The results showed that the elastic modulus and the extracellular matrix increased significantly after OXA treatment (Figure S6B,C, Supporting Information). Together, these findings verify our conclusions that OXA‐resistant GC cells actively remodel the tumor microenvironment by increasing extracellular matrix stiffness.

### 2.6 High Matrix Stiffness Activates RhoA/ROCK1 Signaling Pathway of MSCs

**Figure 6:**
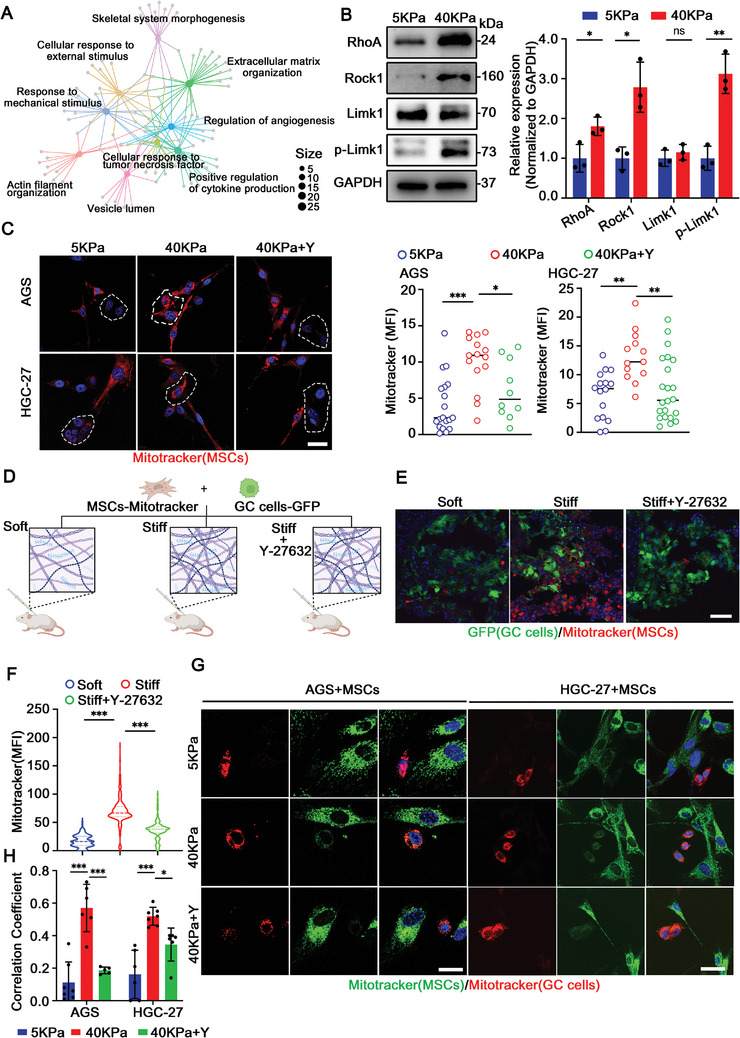
High matrix stiffness activates the RhoA/ROCK1 signaling pathway of MSCs. A) Gene‐Concept Network of pathway enrichment analysis in Vehicle group and OXA group. B) Western blot analysis and quantification of protein levels in RhoA/ROCK1 pathway in MSCs under different conditions (*n* = 3). C) Representative immunostaining images of mitochondria transfer from MSCs to GC cells under different conditions. Mitochondria of MSCs were stained with MitoTracker Red, and the mitochondrial transfer was shown in the white circle. The quantification of MFI of Mitotracker under different conditions (*n* = 3). Scale bar: 20 µm. D) Schematic diagram of MSCs‐GC cells coinjected with low dose collagen I (soft) or high dose collagen I (stiff) in a xenograft model. E) Representative immunostaining images of mitochondrial transfer in each group (soft matrix, stiff matrix, and stiff matrix+Y‐27632). Scale bar: 30 µm. F) Quantification of MFI of MitoTracker Deep Red uptaken by GC cells (*n *= 6 mice group^−1^). G) Representative immunostaining images of mitochondrial fusion under different conditions. Scale bar: 20 µm. H) Quantitative analysis of mitochondrial colocalization (*n* = 3). The data above are presented as mean ± S.D. of three independent experiments. *P*‐values are calculated between two groups was performed using an unpaired *t*‐test, and multiple‐group statistical analysis was performed using one‐way analysis of variance (anova) followed by the Tukey multiple‐comparison test. ns, not significant; *P < 0.05; **P < 0.01; ***P < 0.001.

To explore the potential mechanism by which high matrix stiffness promoted OXA resistance, we analyzed scRNA‐seq data and found that RhoA/ROCK1 pathway related biological phenotypes were obviously enriched (**Figure** **6**A), suggesting that RhoA/ROCK1 pathway might play a key role in MVs formation and mitochondrial transfer. To verify this hypothesis, MSCs‐GC cells were co‐cultured in polyacrylamide hydrogels with varying degrees of stiffness. The results showed that high matrix stiffness activated the RhoA/ROCK1 pathway and promoted mitochondrial transfer, while the inhibitor of ROCK1 (Y‐27632) could block the mitochondrial transfer (Figure 6B,C). At the same time, the conditioned medium of MSCs was extracted and used for GC cell culture under different matrix stiffness. The results showed that high matrix stiffness promoted mitochondrial transfer, while Y‐27632 blocked mitochondrial transfer (Figure S7A–C, Supporting Information).

In order to further explore high matrix stiffness activates the RhoA/ROCK1 signaling pathway of MSCs, we co‐injected MSCs‐GC cells mixed with collagen I into mice to simulate extracellular matrix stiffness in vivo (Figure 6D). Immunofluorescence results showed that Y‐27632 inhibited mitochondrial transfer on high matrix stiffness (Figure 6E,F). In the MSCs‐GC cells coculture system, high matrix stiffness promoted mitochondrial fusion and reduced the levels of mitophagy, thus promoting GC cells survival, while Y‐27632 inhibited this process (Figure 6G,H; Figure S7D–F, Supporting Information). Together, these results indicate that the high matrix stiffness promotes mitochondria to transfer from MSCs to GC cells by activating the RhoA/ROCK1 signaling pathway of MSCs, which reduces the levels of mitophagy of GC cells and promotes the survival of GC cells.

### 2.7 Targeting RhoA/ROCK1 Signaling Pathway Reduces Mitochondrial Transfer and Restores the Tumor‐Killing Effect of OXA

**Figure 7:**
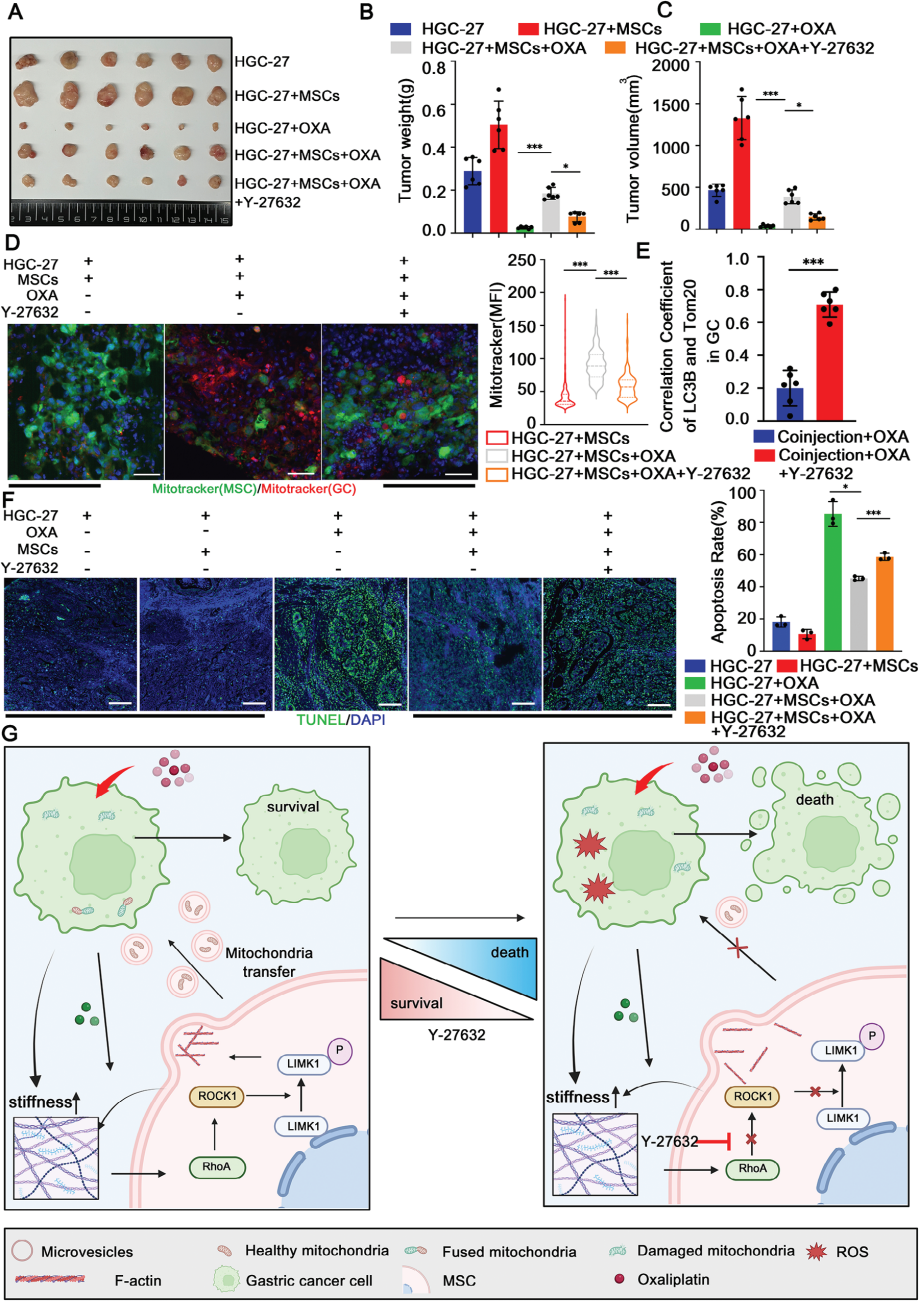
Targeting the RhoA/ROCK1 signaling pathway restores the tumor‐killing effect of OXA. A) Representative images of tumors from mice in each group (HGC‐27, HGC‐27‐MSCs, HGC‐27+OXA, HGC‐27‐MSCs+OXA, HGC‐27‐MSCs+OXA+Y‐27632) (*n* = 6 mice group^−1^). B,C) Statistical analysis of subcutaneous tumor weights and tumor volumes (*n* = 6 mice group^−1^). D) Representative immunostaining images of mitochondrial transfer in each group (HGC‐27‐MSCs, HGC‐27‐MSCs+OXA and HGC‐27‐MSCs+OXA+Y‐27632). Scale bar: 30 µm. Quantification of MFI of Mitotracker in each group (*n* = 6 mice group^−1^). E) Quantification of the levels of colocalization in (D) and correlation coefficient are shown under each condition (*n* = 6 mice group^−1^). F) Representative immunostaining images of TUNEL staining in each group (HGC‐27, HGC‐27+OXA, HGC‐27‐MSCs, HGC‐27‐MSCs+OXA, HGC‐27‐MSCs+OXA+Y‐27632), and quantitative analysis of apoptotic cells was shown on the right (*n* = 6 mice group^−1^). Scale bar: 200 µm. The data above are presented as mean ± S.D. of three independent experiments. *P*‐values are calculated between two groups was performed using an unpaired *t*‐test, and multiple‐group statistical analysis was performed using one‐way analysis of variance (anova) followed by the Tukey multiple‐comparison test. ns, not significant; *P < 0.05; **P < 0.01; ***P < 0.001. G) Schematic illustration of targeting the RhoA/ROCK1 pathway of MSCs to alleviate the OXA resistance. After OXA treatment, GC cells actively remodel the extracellular matrix, increase matrix stiffness, activate the RhoA/ROCK1 signaling pathway of MSCs, and promote mitochondria transfer from MSCs to GC cells. Pharmacological intervention by Y‐27632 reduces by MVs‐dependent mitochondrial transfer, restores the tumor‐killing effect of OXA.

To further investigate whether targeting the RhoA/ROCK1 pathway could enhance the killing effect of OXA on GC cells in vivo. These tumor‐bearing mice were then treated with OXA (5 mg kg^−1^) and Y‐27632 (10 mg kg^−1^) since day ten, and tumors were harvested at the 30th day. Compared with the OXA group, the weight and volume of tumors in the HGC‐27‐MSCs+OXA group significantly increased, while Y‐27632 decreased the weight and volume of tumors (**Figure** **7**A–C), suggesting that MSCs attenuated the OXA killing effect, while Y‐27632 inhibited this process. Furthermore, we examined mitochondrial transfer and the levels of mitophagy by immunofluorescence. The results showed that Y‐27632 inhibited the mitochondria transfer from MSCs to GC cells and increased the levels of mitophagy of GC cells (Figure 7D,E; Figure S7G, Supporting Information). At the same time, we also found that apoptosis was significantly increased in the HGC‐27‐MSCs +OXA+Y‐27632 group (Figure 7F). In all, OXA‐resistant GC cells actively remodel the tumor microenvironment by increasing ECM stiffness. High matrix stiffness activates the RhoA/ROCK1 signaling pathway of MSCs, and drives mitochondria transfer from MSCs to GC cells, and restores the mitochondrial function of GC cells, thereby reducing the mitophagy levels of GC cells, and improving cell survival. Targeting the matrix stiffness‐related signaling pathways effectively alleviates OXA resistance and improves OXA therapeutic efficacy (Figure 7G).

## 3 Discussion

ECM stiffness is a risk factor for tumor progression and resistance. This article focuses on the mechanism of ECM stiffness in OXA resistance. We analyzed OXA‐resistant GC samples using single‐cell sequencing for the first time and found that OXA‐resistant GC cells actively remodel the TME by increasing ECM stiffness. High matrix stiffness promotes mitochondria to transfer from MSCs to GC cells via MVs. MSCs‐derived mitochondria fuse with mitochondria of GC cells, reducing the levels of mitophagy in GC cells and promoting GC cell survival. We verify the role of tumor mechanical microenvironment on OXA resistance through in vitro and in vivo experiments.

The tumor microenvironment plays an important role in tumor progression and drug resistance.^[^ ^7a^ ^]^ ECM includes highly organized interactions of fibrous molecules, proteoglycans, glycoproteins, glycosaminoglycans, and other macromolecules. ECM stiffness is a hallmark of solid tumors, and excessive collagen and hyaluronic acid are responsible for its stiffness.^[^ ^7d^ ^]^ We analyzed OXA‐resistant samples using single‐cell sequencing and found that there were two types of cells that promote matrix stiffness increasing: GC cells and MSCs. We further used coculture assays, and found that MSCs in MSCs‐GC cells coculture system expressed higher ECM‐related genes than MSCs in monoculture after OXA treatment. The results indicate that GC cells could directly regulate MSCs, promote ECM accumulation, and actively remodel the tumor microenvironment.

In recent years, many researches have been studied in the mechanisms of high matrix stiffness in chemotherapy resistance,^[^ ^7^, ^26^ ^]^ and the main mechanisms of drug resistance are focused on the following points:^[^ ^26^ ^]^ 1) The stiffness matrix forms a physical barrier for drugs to penetrate into tumor tissue. 2) The stiffness matrix also compresses microvasculature, making it difficult for drugs to enter the core tumor tissue through the vascular system. 3) The stiffness matrix stiffness induces hypoxia in the tumor microenvironment, leading to the disorganization of intratumoral microvessels. 4) The stiffness matrix is involved in the transformation of tumor cells into cancer stem cells. In our study, scRNA‐seq data showed that the RhoA/ROCK1 pathway was obviously enriched in OXA‐resistant GC samples, suggesting that RhoA/ROCK1 may play a key role in tumor matrix stiffness. We further found that the RhoA/ROCK1 signaling pathway was activated on the high matrix stiffness, and promoted mitochondria to transfer from MSCs to GC cells. Furthermore, we demonstrated in vivo experiments that the high matrix stiffness promoted more mitochondrial transfer, and blocking mechanical‐related pathways by Y‐27632 significantly inhibits mitochondrial transfer from MSCs to GC cells. Therefore, for the first time, we speculate that mitochondrial transfer is mediated by matrix stiffness.

Hypoxia and vigorous metabolism are common features of malignant tumors, which require much more energy than normal cells.^[^ ^27^ ^]^ However, mitochondrial structural abnormalities are prevalent in various cancers, resulting in severe damage to cellular aerobic respiratory function.^[^ ^28^ ^]^ Therefore, in order to meet the rapid proliferation of tumors themselves, it is necessary to timely replenish and repair the mitochondria of tumor cells.^[^ ^28^ ^]^ Our analysis of single‐cell sequencing data showed that these DEGs are enriched in mitochondrial pathways. And the role of mitochondria in drug resistance has also been a hot topic in recent years. For instance, researchers have shown that drug‐resistant cancer cells present high levels of antioxidant factors that counteract the increase in  ROS induced by chemotherapy.^[^ ^29^ ^]^ Another study showed that resistant‐cancer cells can transmit signals from damaged mitochondria to the nucleus, resulting in altered expression of nuclear‐encoded genes, which may facilitate metabolic reprogramming and cell adaptation.^[^ ^30^ ^]^ In addition, studies have shown that changes in mtDNA of drug‐resistant cancer cells can lead to chemotherapy resistance.^[^ ^31^ ^]^ In our study, we found that the mechanism of OXA‐resistant in GC is to restore the mitochondrial function of GC cells and maintain mitochondrial homeostasis by reducing mitochondrial membrane potential and ROS production after mitochondrial transfer. In addition, we also found that GC cells reduce the killing effect of OXA by decreasing the levels of mitophagy in GC cells.

Intercellular communication is a fundamental process by which every multicellular organism shares cytoplasmic components. Mitochondrial transfer was first observed in 2006, and Spees et al. demonstrated that the transfer of functional mitochondria from human stem cells to recipient mitochondria‐deprived cells can restore mitochondrial respiration.^[^ ^32^ ^]^ Studies in the field of cancer have also found that a variety of cell types in the tumor microenvironment can regulate the state of tumor mitochondria by directly contact with tumor cells^[^ ^32^ ^]^ or indirectly secreting soluble factors or various extracellular vesicles to tumor cells.^[^ ^33^ ^]^ In the past two decades, it has been discovered that mitochondrial transfer occurs through TNTs, MVs, and gap junctions, which may be one of the mechanisms of tumor chemotherapy resistance.^[^ ^34^ ^]^ In our study, we found that mitochondria were enriched in OXA‐resistant gastric cancer cells, and the increase in mitochondria within tumor cells was not endogenous synthesis, suggesting that mitochondria might originate from the tumor microenvironment. Then we co‐cultured MSCs, PBMCs, and HUVECs with GC cells in vitro, and found that MSCs were the main cells that transferred mitochondria to GC cells. TNT is reported to be the main mitochondrial transfer pathway, but in our research, we used three inhibitors and found that after OXA treatment, mitochondria transferred from MSCs to GC cells by MVs. Although mitochondrial transfer has been observed in a variety of pathological conditions, the regulatory factors mediating mitochondrial transfer remain unclear. Anurag Agrawal et al. demonstrated that mitochondrial RhoA‐GTPase Miro1 regulated mitochondrial intercellular movement.^[^ ^35^ ^]^ Kazuhide Hayakawa et al. demonstrated that the astrocytic release of extracellular mitochondrial particles was mediated by a calcium‐dependent mechanism involving CD38 and cyclic ADP ribose signaling.^[^ ^33^ ^]^ In our study, we demonstrated for the first time that high matrix stiffness was an important factor in promoting mitochondrial transfer. OXA‐resistant GC cells actively remodelled the extracellular matrix, and high matrix stiffness activated the RhoA/ROCK1 signaling pathway of MSCs, thus promoting the mitochondria to transfer from MSCs to GC cells, so as to repair the mitochondrial function, reduce the killing effect of OXA. As the same time, we also found that mitochondrial transfer in OXA‐resistant GC cells was unidirectional, from MSCs to GC cells.

In all, we find that OXA resistance in gastric cancer is correlated with matrix stiffness, revealing that matrix stiffness can be used as a way to diagnose chemotherapy resistance. In terms of the mechanism of OXA resistance, we find that high matrix stiffness promotes the mitochondria to transfer from MSCs to GC cells, which helps tumor cells survive, thereby leading to chemotherapy resistance. Targeting the mechanical‐related RhoA/ROCK1 pathway can inhibit mitochondrial transfer and alleviate chemotherapy resistance. Therefore, we propose that in the treatment of gastric cancer, the combination of RhoA/ROCK1 inhibitors may enhance the tumor‐killing effect of OXA.

## 4 Experimental Section

### Patients and Samples

Five patients with advanced gastric cancer (clinical stage: cT3‐4N+M0) who visited the Department of Gastric Surgery, Sun Yat‐sen University Cancer Center (SYSUCC) from October 2023 to December 2023 were selected. All five patients underwent chemotherapy with OXA 130 mg m^−2^ as the main regimen for 3–4 courses (21 days per course) prior to surgery. And the postoperative specimens were classified as tumor regression grade (TRG) 3: poor pathological response, a large number of residual cancer cells, and no obvious tumor regression. These patients were defined as OXA‐resistant. Samples of these five patients were collected before and after OXA chemotherapy for comparative analysis of single‐cell sequencing. Correspondingly, the gastroscopy samples before chemotherapy were taken as Vehicle group, and the surgical samples after OXA chemotherapy were taken as OXA‐resistant group, referred to as OXA group. This study was reviewed and approved by the Medical Ethics Committee of Sun Yat‐sen University Cancer Center (Accreditation number: SL‐B2022‐751‐02, SL‐B2022‐038‐03). All patients provided written informed consent in accordance with the principles of the Declaration of Helsinki.

### Cell Preparation

After harvested, human tumor specimens were washed in ice‐cold RPMI 1640 (Gibco) and dissociated using Tissue Dissociation Reagent A Pro (Seekone K01801‐30) from SeekGene as instructions. DNase I (Sigma) treatment was optional according to the viscosity of the homogenate. Cell count and viability was estimated using fluorescence Cell Analyzer (Countstar Rigel S2) with AO/PI reagent after removal erythrocytes (Solarbio) and then debris and dead cells removal was decided to be performed or not (Milteny). Finally, fresh cells were washed twice in the RPMI 1640 and then resuspended at 1 × 10^6^ cells per mL in 1 × PBS (Gibco) and 0.04% bovine serum albumin (BSA; Beyotime).

### Single Cell RNA‐Seq Library Construction and Sequencing

Single‐cell RNA‐Seq libraries were prepared using SeekOne Digital Droplet Single Cell 3′ library preparation kit (SeekGene). Briefly, appropriate number of cells were mixed with reverse transcription reagent and then added to the sample well in SeekOne chip. Subsequently Barcoded Hydrogel Beads (BHBs) and partitioning oil were dispensed into corresponding wells separately in chip. After emulsion droplet generation reverse transcription were performed at 42 °C for 90 min and inactivated at 80 °C for 15 min. Next, cDNA was purified from broken droplet and amplified in PCR reaction. The amplified cDNA product was then cleaned, fragmented, end repaired, A‐tailed and ligated to sequencing adaptor. Finally, the indexed PCR were performed to amplified the DNA representing 3′poly A part of expressing genes which also contained Cell Barcode and Unique Molecular Index. The indexed sequencing libraries were cleanup with SPRI beads, quantified by quantitative PCR (KAPA Biosystems) and then sequenced on illumina NovaSeq 6000 with PE150 read length.

### Quality Control

For quality control, cells detected genes <500 or >6000 were filtered out. In addition, cells with over 15% of mitochondria‐derived UMI counts were considered to be low‐quality cells and were also excluded. Finally, 74 367 single cells were left for downstream analysis.

### Copy Number Variation (CNV) Analysis

The CNV levels were evaluated through the normalization of scRNA‐seq gene expression arrays with the inferCNV R package.^[^ ^36^ ^]^ For each patient, epithelial cells were considered as the putative tumor epithelium dataset. Immune cells were considered as the reference dataset. The final CNV profiles were obtained by denoising, while sex chromosomes were excluded to estimate and identify malignant cells.

### Canonical Correlation Analysis (CCA), Dimensionality Reduction, and Clustering

After quality control and filtering, library‐size normalization to each cell was performed by NormalizeData of Seurat (version 4.0.0).^[^ ^37^ ^]^ The variable genes were calculated by FindVariableGenes. Then, all libraries were combined together using FindIntegrationAnchors and IntegrateData, and using ScaleData to regress out the variability of the numbers of UMIs. Then the RunPCA and RunUMAP was used to reduce dimensions. FindClusters was used to cluster cells using the 20 dims at a resolution of 0.8.

### Signature Score Calculation

Seurat Function AddModuleScore was used to combine the expression of gene list. The data set was downloaded from Msigdb database.

### Differentially Expressed Genes (DEGs) Analysis

The FindMarkers function was used to identify DEGs across the conditions, and the default Wilcoxon rank test was used. Genes were ranked by absolute log2 fold‐change (log2FC), and those with *P*‐values > 0.05 (adjusted for multiple comparisons), min.pct <0.05, or log2FC <0.1 were removed.

### Enrichment Analysis

Gene Ontology enrichment analysis of DEGs was implemented by the clusterProfiler R package.^[^ ^38^ ^]^ GO terms with corrected P value less than 0.05 were considered significantly enriched by DEGs. ClusterProfiler R package was used to test the statistical enrichment of DEGs in GO pathways.

### Cell–Cell Interactions

Cell Chat R package and iTALK R package were used to detect ligand‐receptor interactions and predict communications among different cell types. These packages were publicly available repository of curated receptors and ligands and their interactions.

### Animal Study

The mouse experiments were carried out according to protocols approved by the Institutional Animal Care and Use Committee (IACUC) of the Shenzhen Top Biotech Co., Ltd (Accreditation number: TOP‐IACUC‐2023‐0269). Mice were housed in the University of Top biotech Biotechnology Co., Ltd. (Shenzhen, China) under specific pathogen‐free (SPF) conditions at 23 ± 2 °C ambient temperature with 40% humidity and a 12 h light/dark cycle (8 am on and 8 pm off).

For establishing a syngeneic mouse GC model in immunodeficient NOD*‐scid* common γ chain–deficient (NSG) mice, mice were randomly assigned subjects to control group and treatment groups, and GC cells (3 × 10^6^ cells in 100 µL medium) and/or MSCs (1 × 10^6^ cells) were subcutaneously injected into the right/left flanks of 8‐week‐old male mice. According to different experimental requirements, the corresponding concentrations of compounds used were as follows: OXA (5 mg kg^−1^); Y‐27632 (10 mg kg^−1^). Animal experiments designed according to the needs include: mitochondrial transfer in vivo, and the therapeutic effect of Y‐27632 in vivo.

To investigate the therapeutic effect of Y‐27632 on matrix stiffness, Matrigel was mixed with various concentrations Collagen I (CoL1) to form different matrix stiffness. Matrgel solution (6 mg mL^−1^ Matrigel in 100 µL serum‐free medium), soft matrix (6 mg mL^−1^ Matrigel and 3.5 mg mL^−1^ CoL1 in 100 µL serum‐ free medium) and stiff matrix (6 mg mL^−1^ Matrigel and 70 mg mL^−1^ CoL1 in 100 µL serum‐free medium), respectively. The suspended GC cells and MSCs were subcutaneously injected into the upper right/left flank region of 8 week‐old male mice, and the growth of subcutaneous tumors was observed. When tumors reached a volume of 100–150 mm, OXA was administered every three days and Y‐27632 was administered daily. Mice were treated with OXA and/or Y‐27632 for 15 days. Mice were weighed every 2 days. Tumors were measured using a digital caliper and the tumor volume was calculated by the formula: (width)^2 ^× length/2. The mice were euthanized before the longest dimension of the tumors reached 2.0 cm, as required by IACUC.

### Cell Culture and In Vitro Coculture System

HGC‐27, AGS, HUVECs were purchased from the Cell Bank of the Chinese Academy of Sciences (Shanghai). The cell culture media were RPMI 1640, and high‐glucose DMEM (Gibco), supplemented with fetal bovine serum (FBS; Gibco), penicillin (Sigma), and streptomycin (Sigma). PBMCs isolated from a health volunteer. Peripheral blood (10 mL) was collected using EDTA anticoagulation tubes (BD) and processed immediately. According to the instructions, the steps were as follows: Diluted whole blood with 10 mL PBS (Volume ratio 1:1). Added 10 mL lymphocyte separation solution Ficoll (Merch) and centrifuge at 400 g for 30 min. PBMCs were collected at the junction of the plasma layer and lymphocyte separation fluid, and sample was transferred into a new centrifuge tube. MSCs was donated by the Center for Stem Cell Biology and Tissue Engineering, Key Laboratory for Stem Cells and Tissue Engineering, Sun Yat‐sen University. GC cells were plated in direct contact with PBMCs^[^ ^39^ ^]^ or HUVECs^[^ ^40^ ^]^ or MSCs^[^ ^41^ ^]^ at a 1:20, 1:4, 3:1 ratio. 18‐α‐GA, dynasore, and cytochalasin D were purchased from Sigma‐Aldrich and were used concentrations of 50, 50, and 0.2 µm, respectively. All the cells were cultured in a humidified 5% CO_2_ – 95% air environment at 37 °C.

### Preparation of Polyacrylamide Gels of Different Stiffness Levels

Polyacrylamide hydrogel was obtained with 40% w/v acrylamide (Acr; Diamond) + 2% w/v bis‐acrylamide (Bis; Yuanye), adding the coagulant 10% ammonium persulfate (APS; Sangon Biotech) and tetramethylethylenediamine (TEMED; PHYGENE). Different stiffness was obtained by changing the final concentration of Acr/Bis, which expected stiffness of 5 and 40 KPa according to previous article.^[^ ^42^ ^]^ Acr (40%), 2% Bis in a HEPES‐buffered solution (pH 8), 10% APS (1/100 volume) and TEMED (1/100 volume) were mixed and polymerized to make 1 mm‐thick uniform flat PAGE gels with different stiffness levels. Then, a gel was cut into squares that fit the size of culture dish. The coverslips were washed twice for 20 min each in PBS and 500 µL of heterobifunctional sulfosuccinimidyl 6‐(4′‐azido‐2′‐nitrophe‐nylamino) hexanoate was added and photoactivated for 60 min with UV light. After being rinsed with a PBS solution, coated with 2 mL fibronectin (100 µg mL^−1^), overnight at 37 °C, UV disinfection for 1 h the next day, and rinsed before cells seeding.

### Conditioned Medium

Before the experiment, CFSE labeled GC cells and MitoTracker Red labeled MSCs. After 24 h, conditioned medium (CM) of MSCs was collected in tubes and centrifuged for 10 min at 300 g, 4 °C, followed by 0.45 µm filtration. GC cells were cultured with different matrix stiffness, supplemented with conditioned medium (10% FBS, 1% PS) and kept at 37 °C at least 24 h.

### Mitochondrial Isolation and Transfer

Mitochondria were isolated according to the Cell Mitochondria Isolation Kit (Beyotime) manufacturer's instruction. The isolated mitochondria were suspended in mitochondrial suspension buffer (pH 7.0) at a concentration of 1 mg mL^−1^. Prior to mitochondrial transfer, recipient cells (GC cells) prelabeled with CFSE were harvested from culture flasks, and 1 × 10^5^ cells were transferred to a microcentrifuge tube. GC cells were suspended in 100 µL of RPMI 1640 and kept on ice for transfer. The mitochondrial suspension (in 100 µL of mitochondrial suspension buffer) was added slowly to each tube of GC cells suspended in 100 µL of RPMI 1640. The microcentrifuge tubes were centrifuged at 1500 × *g* for 5 min at 4 °C, then the cells in the centrifuge tube were extracted and transferred to the six‐well plate for implantation and placed in a 37 °C incubator. The next day, cells were harvested for further testing.

### Immunofluorescence Staining (IF)

Samples were fixed in 4% PFA fix solution (Biosharp) for 15 min, and incubated with PBS containing 0.25% Triton X‐100 ((Diamond) for 10 min at 4 °C. Then the cells were blocked with 10% BSA for 30 min. Following blocking, the cells were incubated with primary antibodies listed in Table S1 (Supporting Information) overnight at 4 °C. After washing, the cells were incubated with secondary antibodies for 60 min at 37 °C and visualized under Zeiss 880 Laser Scanning Confocal Microscope with Airyscan (ZEISS) and highspeed spinning disk confocal microscope (Andor Dragonfly 202 Imaging System).

### Immunohistochemical Staining (IHC)

Sections of the paraffin‐embedded tumor samples were kept at 60 °C for 3 h in the oven and then followed by dewaxing with xylene and hydrating with an ethanol gradient (100–70%). After soaking in 3% H_2_O_2_ for 30 min, the slides were rinsed with PBS and incubated with the primary antibody overnight at 4 °C. And then the slides were rinsed and incubated with the corresponding secondary antibody for 30 min followed by DAB and hematoxylin staining, respectively. The primary and secondary antibodies used can be found in the Table S1 (Supporting Information). The slides were then examined and photographed using an Olympus BX53 microscope. The DAB staining was analyzed by ImageJ software.

### MitoTracker Staining

MitoTracker Red (Invitrogen) and MitoTracker Deep Red (Invitrogen) were used to label mitochondria. PBMCs, HUVECs or MSCs or GC cells were incubated with 200 nmdf MitoTracker Red in culture media for 30 min at 37 °C. Excess of the dye was washed out with PBS. Then, 1 day later, stained cells were then seeded for monoculture and coculture. MFI of MitoTracker was measured from three independent experiments.

### Annexin V/PI Flow Cytometry Analysis

Cells were harvested by centrifugation and then stained with Annexin V/PI (BioLegend) according to the manufacturer's instruction. The apoptotic population was immediately evaluated by flow cytometry (CytoFLEX). The percentages of apoptotic cells were analyzed and graphed.

### TMRE Staining

The mitochondria‐specific dye, TMRE was determined to measure cells mitochondrial potential changes during apoptosis. AGS and HGC‐27 were resuspended in their respective complete media and incubated with TMRE (Beyotime) for 20 min at 37 °C. At the end of incubation, cells were washed in Hank's balanced salt solution and re‐suspended in the flow analysis buffer, D‐PBS substituted with 0.2% BSA, and evaluated by flow cytometry (Beckman), and then analyzed using FIJI software.

### Mitochondrial ROS Assessment

The levels of mitochondrial ROS were detected using Reactive Oxygen Species Assay Kit (Beyotime), and fluorescent intensity was measured by flow cytometry (Beckman). MFI of MitoSOX was measured from three independent experiments.

### Measurement of ATP Content

To measure the release of ATP, the supernatant from each group was collected. ATP was quantified using an ATP assay kit (Beyotime) according to the manufacturer's protocols on a microplate reader (Biotek SynergyH1).

### Cell Viability Assay using by Calcein‐AM/PI Staining

After different treatments, the cells were washed once with PBS, and then the cells were stained with calcein‐AM and PI per well at 37 °C for 30 min. Then, the cells were washed with PBS three times to remove excess calcein‐AM/PI. The images of the cells were acquired immediately and analyzed by using a fluorescence microscope (Leica DMi8). The percentage of positive cells was counted and the average fluorescence intensity was assessed with FIJI software.

### In Vitro Live Imaging Analysis

To better observe mitochondrial transfer, we labeled MSCs mitochondria with MitoTracker Red. For time‐lapse imaging, live cells were acquired using a Lionheart FX automated microscope (Biotek) at 0 to 90 mins time points. The brightfield‐fluorescence merged images were captured for each time of view. The presented movie was acquired using the same microscopy settings and with cells maintained at 37 °C.

### Atomic Force Microscopy (AFM)

The patient and mouse tumor specimens were embedded with optimal cutting temperature and made into frozen sections. The height and phase signals of the aortic frozen tissue slice were recorded by AFM which was used to measure the Young's modulus. AFM software NanoScope version 1.4 was then used to analyze optical, structural, and electrical characterization of the acquired images.

### Co‐Immunoprecipitation and Immunoblot Ayalysis

MSCs was transfected with lentivirus containing HA‐tag Annaxin‐A1 (MVs marker). The supernatant of each group extracted the MVs and used the HA‐tag antibody to co‐immunoprecipite to detect the content of MSCs‐derived MVs. Cells were washed twice with cold PBS and lysed in 1X RIPA buffer (Solarbio) with 30 min ice‐bath and centrifuged at 13 000 g for 15 min at 4 °C to remove cell debris. The total cell protein was located in the supernatant lysate. The total protein concentration was assessed by BCA Protein Assay Kit (Thermo Scientific). Equal amount of protein was resolved by SDS polyacrylamide gel electrophoresis (EpiZyme). Proteins were electrotransferred to a 0.45 µm pore‐sized polyvinylidene difluoride membrane (PVDF; Millipore). The membranes were incubated with blocking buffer (5% skimmed milk in TBS‐T buffer) for 1 h. After blocking, the target proteins were immunoblotted with specific antibodies. The primary and secondary antibodies used can be found in the Table S1 (Supporting Information). The protein signals were detected by the ChemiDoc imaging system (Bio‐Rad). Bands from at least three independent experiments were quantified using ImageJ software.

### RNA Isolation and Quantitative Real‐Time PCR (qRT‐PCR)

Total RNA was extracted from cells using TRIzol reagent (Life Technologies) according to the manufacturer's protocol. Quantification was performed on a NanoDrop 8000 spectrophotometer (Thermo scientific), and 500 ng of total RNA per sample was reverse transcribed into complementary DNA (cDNA) using PrimeScript RT reagent Kit (Takara). cDNA was amplified using SYBR kits (Takara) on Bio‐rad CFX96 Touch Real‐Time PCR System. All samples were run in duplicate, and the results were normalized to glyceraldehyde‐3‐phosphate dehydrogenase (GAPDH) as a relative mRNA level. The primers used for real‐time PCR can be found in the Table S2 (Supporting Information).

### Statistical Analysis and Reproducibility

All data were obtained from at least three independent experiments and expressed as mean ± SD. Sample sizes were indicated in the figure legend. Statistical analysis between two groups was performed using an unpaired *t*‐test. Multiple‐group statistical analysis was performed using one‐way analysis of variance (anova) followed by the Tukey multiple‐comparison test. Prism software (GraphPad) was used for data analysis. A two‐sided *P*‐value of less than 0.05 was considered statistically significant. Significance levels were defined as *P *< 0.05 (*), *P *< 0.01 (**), *P *< 0.001 (***).

## Conflict of Interest

The authors declare no conflict of interest.

## Author Contributions

X.H., L.Z., N.W., and B.W.Z. contributed equally to this work. J.C.W. designed and supervised this study. B.W.Z. managed patients. X.H., L.Z., N.W., X.X.W., C.Y.Z., and J.F.H. performed experiments, analyzed data, acquired and provided facilities, and cell lines, etc. X.H., J.F.H., C.Y.Z., and Y.H.R. analyzed and interpreted of data (e.g., statistical analysis, biostatistics and computational analysis). J.C.W., X.H., L.Z., and N.W. wrote, reviewed and/or revised of the manuscript. N.W., Y.L.H., P.X., and J.C.W. financial supported.

## Supporting information



Supporting Information

Supplemental Table 1

## Data Availability

The data that support the findings of this study are available from the corresponding author upon reasonable request.
