# Supplementary material for: Gastric Cancer Actively Remodels Mechanical Microenvironment to Promote Chemotherapy Resistance via MSCs‐Mediated Mitochondrial Transfer
Source: Adv Sci (Weinh). 2024 Oct 11;11(47):2404994. doi: 10.1002/advs.202404994 (PMC11653701; doi:10.1002/advs.202404994)
Supplement: Supplementary file 1 — Supporting Information [file ADVS-11-2404994-s001.docx]

**Gastric Cancer Actively Remodels Mechanical Microenvironment to Promote Chemotherapy Resistance via MSCs-mediated Mitochondrial Transfer**

*Xin He, Li Zhong, Nan Wang, Baiwei Zhao, Yannan Wang, Xinxiang Wu, Changyu Zheng, Yueheng Ruan, Jianfeng Hou, Yusheng Luo, Yuehan Yin, Yulong He,^*^ Andy Peng Xiang,^*^ Jiancheng Wang^*^*

Supporting Information


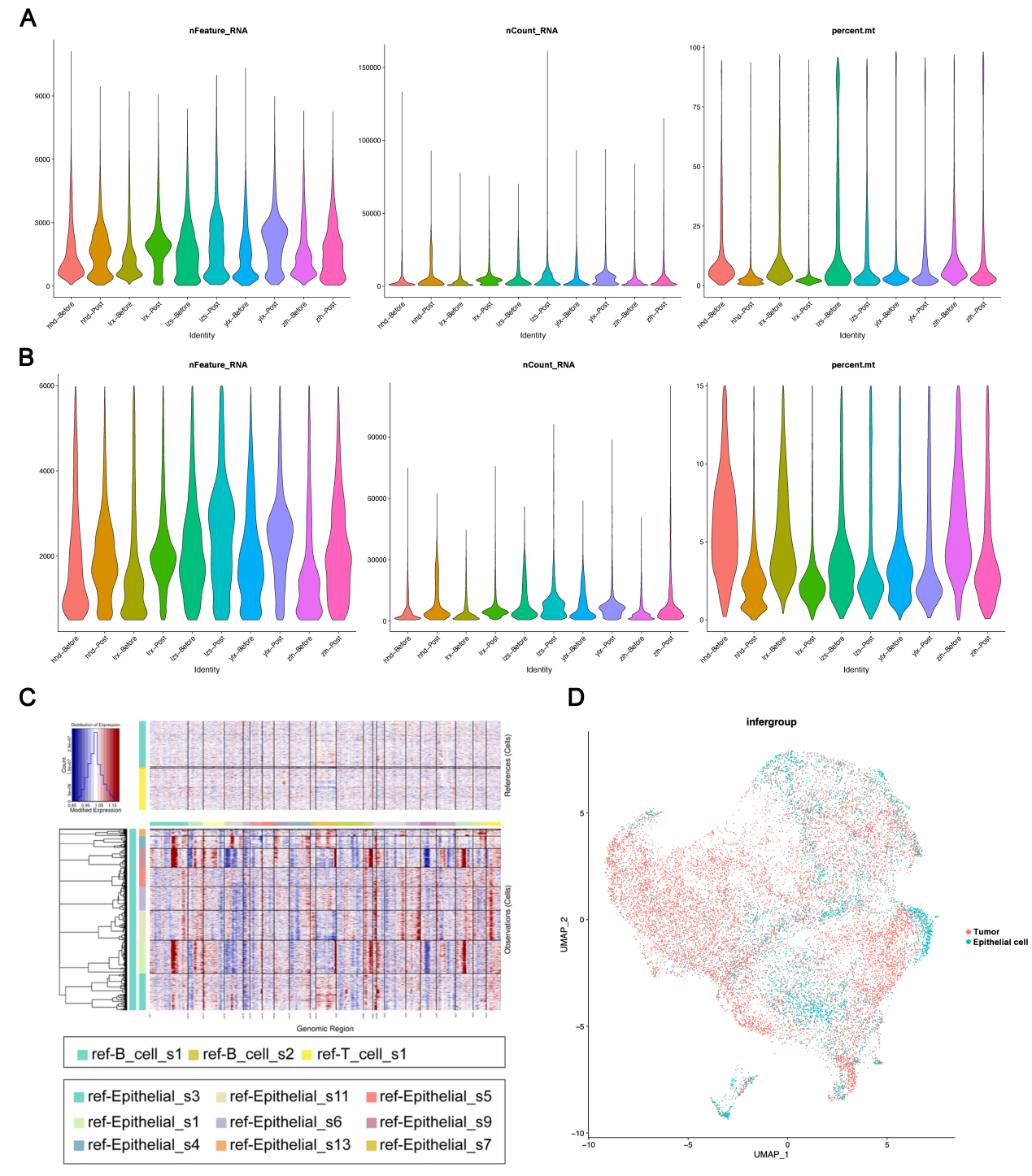


**Figure S1.** Single-cell RNA sequencing in OXA-resistant GC cells.

Violin plot showed the number of detected genes, unique molecular identifier (UMI) counts, and the ratio of mitochondrial-derived UMI counts. Cells with detected genes <500 or >6000, or over 15 % mitochondrial-derived UMI counts were filtered out. (A) Violin plot showed before quality control. (B) Violin plot showed after quality control. (C) Heatmap showed large-scale CNV profile of epithelial cell. Red and blue colors represent chromosomes amplification and deficiency respectively. T cell and B cell were defined as reference cells. (D) Epithelial cell cluster were divided into tumor cells and normal epithelial cells according to inferCNV results.


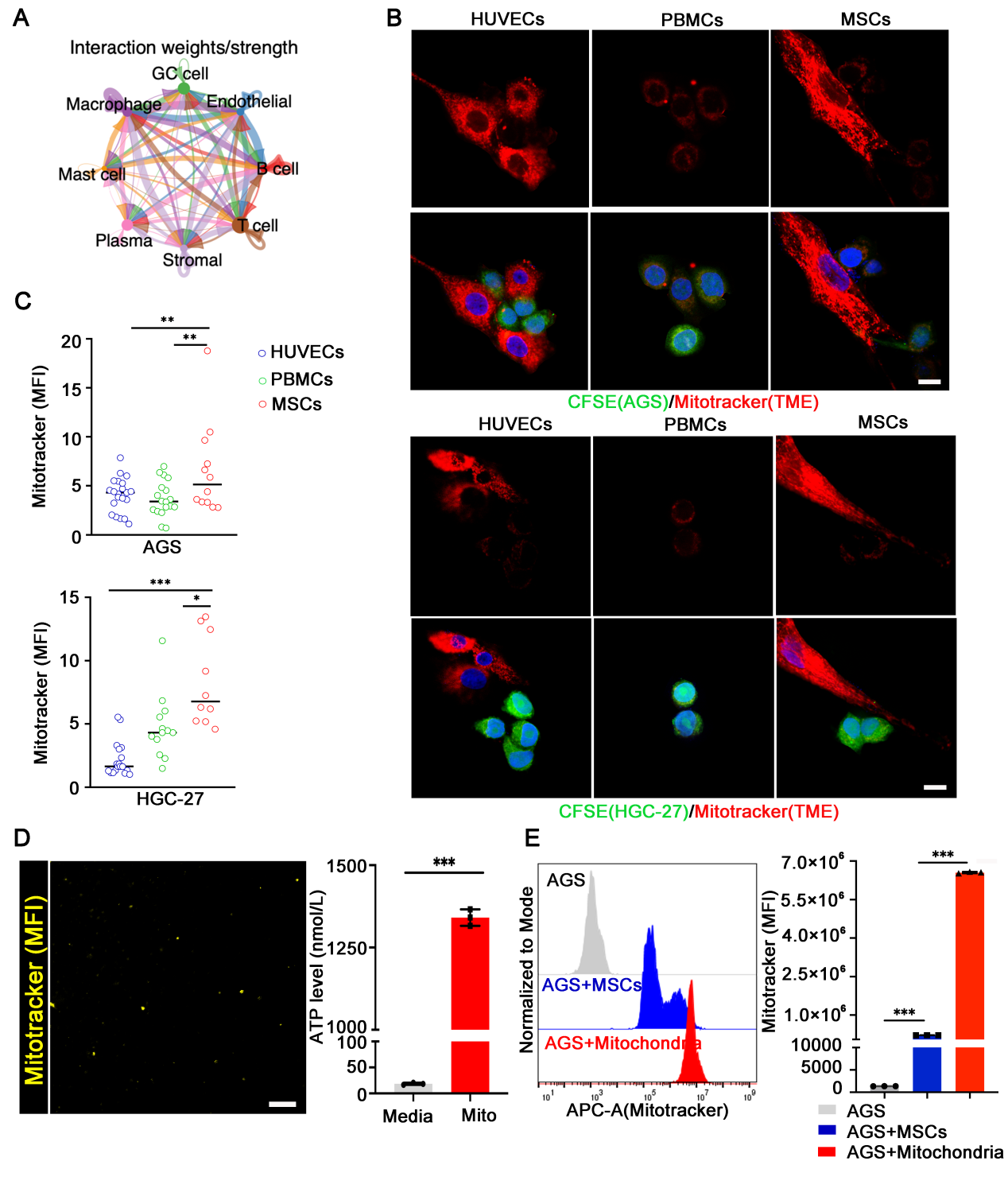


**Figure S2.** MSCs are mainly cells to transfer mitochondria to GC cells.

1. Differences in the strength of interactions of various cell types in OXA-resistant group. The thicker the line, the stronger the interaction. (B) Representative immunostaining images of exogenous mitochondria uptaken by GC cells. CFSE-labeled GC cells were cocultured with MitoTracker Deep Red-labeled MSCs or PBMCs or HUVECs. Scale bar: 20 μm. (C) Quantification of MFI of MitoTracker Deep Red uptaken by GC cells was analyzed (n= 3-4). (D) Confocal microscopic image of mitochondria derived from MSCs. Measurement of the intracellular ATP levels in media or MSCs-derived mitochondria (n = 3). Scale bar: 20 μm. (E) Flow cytometry analysis of Mitotracker in each group (AGS, AGS-MSCs, and AGS+Mitochondria). Quantification of MFI of each group was analyzed and graphed (n = 3). The data above are presented as mean ± S.D. of three independent experiments. *P*-values are calculated between two groups was performed using an unpaired *t*-test, and multiple-group statistical analysis was performed using one-way analysis of variance (anova) followed by the Tukey multiple-comparison test. ns, not significant; **P*<0.05; ***P*<0.01; ****P*<0.001.


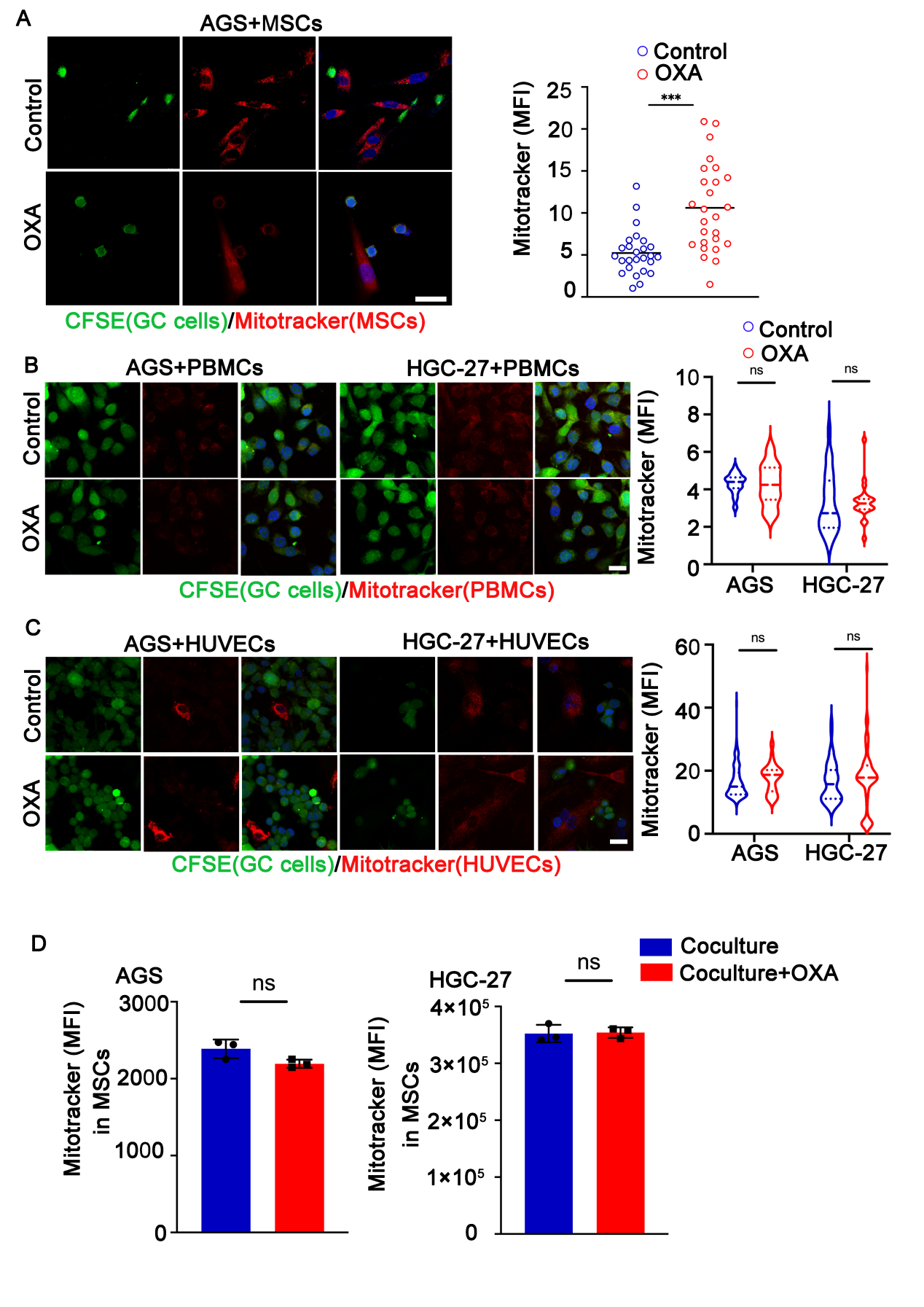


**Figure S3.** After OXA administration, MSCs transfer mitochondria to GC cells.

(A-C) Representative immunostaining images of mitochondrial transfer in each group. CFSE-labeled GC cells were cocultured with MitoTracker Deep Red-labeled MSCs, PBMCs or HUVECs. Scale bar: 30, 20, 20 μm, respectively. Quantification of MFI of MitoTracker Deep Red uptaken by GC cells was analyzed and graphed (n = 3). (D) Quantification of MFI of MitoTracker Deep Red uptaken by MSCs in each group (MSCs-GC cells and MSCs-GC cells+OXA) (n = 3). The data above are presented as mean ± S.D. of three independent experiments. *P*-values are calculated between two groups was performed using an unpaired *t*-test, and multiple-group statistical analysis was performed using one-way analysis of variance (anova) followed by the Tukey multiple-comparison test. ns, not significant; **P*<0.05; ***P*<0.01; ****P*<0.001.


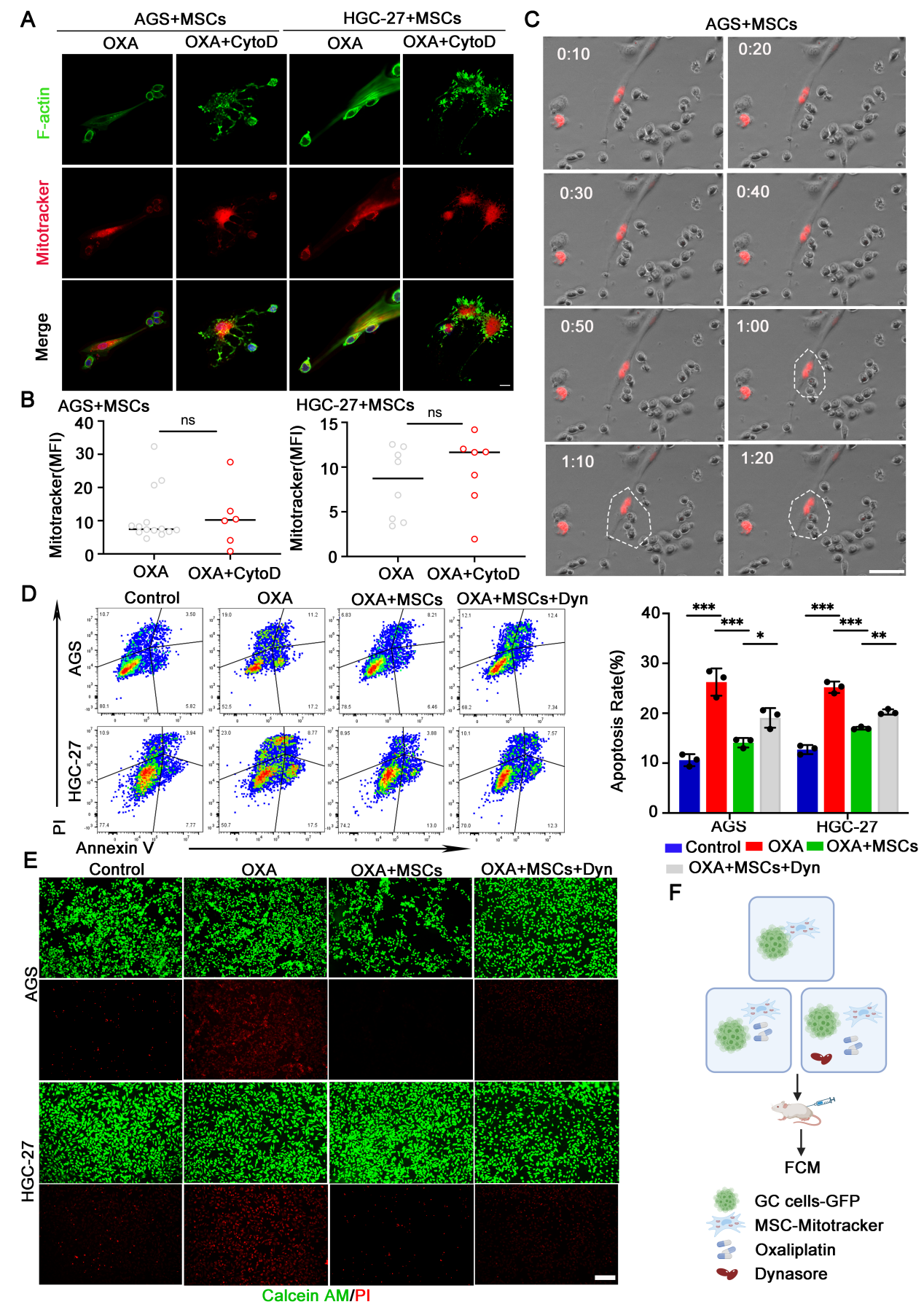


**Figure S4.** MSCs transfer mitochondria to GC cells via microvesicle (MVs).

1. Representative images of mitochondrial transfer in each group (MSCs-GC cells+OXA and MSCs-GC cells+OXA+CytoD). Scale bar: 20 μm. (B) Quantitative analysis of the mitochondrial transfer levels in (A) (n = 3-4). (C) Representative living cells tracing images of mitochondrial transfer in AGS-MSCs coculture system after OXA treatment for 24 hours. MSCs were labeled with MitoTracker Deep Red before cocultured with AGS cells. Scale bar: 50 μm. (D) The apoptosis of GC cells in each group (GC cells, GC cells+OXA, GC cells-MSCs+OXA, MSCs-GC cells+OXA+Dynasore) was analyzed by flow cytometry. Quantitative analysis of GC cells apoptosis rate shown on the right (n = 3). (E) Representative images in each group using calcein-AM/PI staining (GC cells, GC cells+OXA, MSCs-GC cells+OXA, and MSCs-GC cells+OXA+Dynasore). Scale bar: 100 μm. (F) Schematic diagram of therapeutic trials for xenograft models (n = 6 mice/group). The data above are presented as mean ± S.D. of three independent experiments. *P*-values are calculated between two groups was performed using an unpaired *t*-test, and multiple-group statistical analysis was performed using one-way analysis of variance (anova) followed by the Tukey multiple-comparison test. ns, not significant; **P*<0.05; ***P*<0.01; ****P*<0.001.


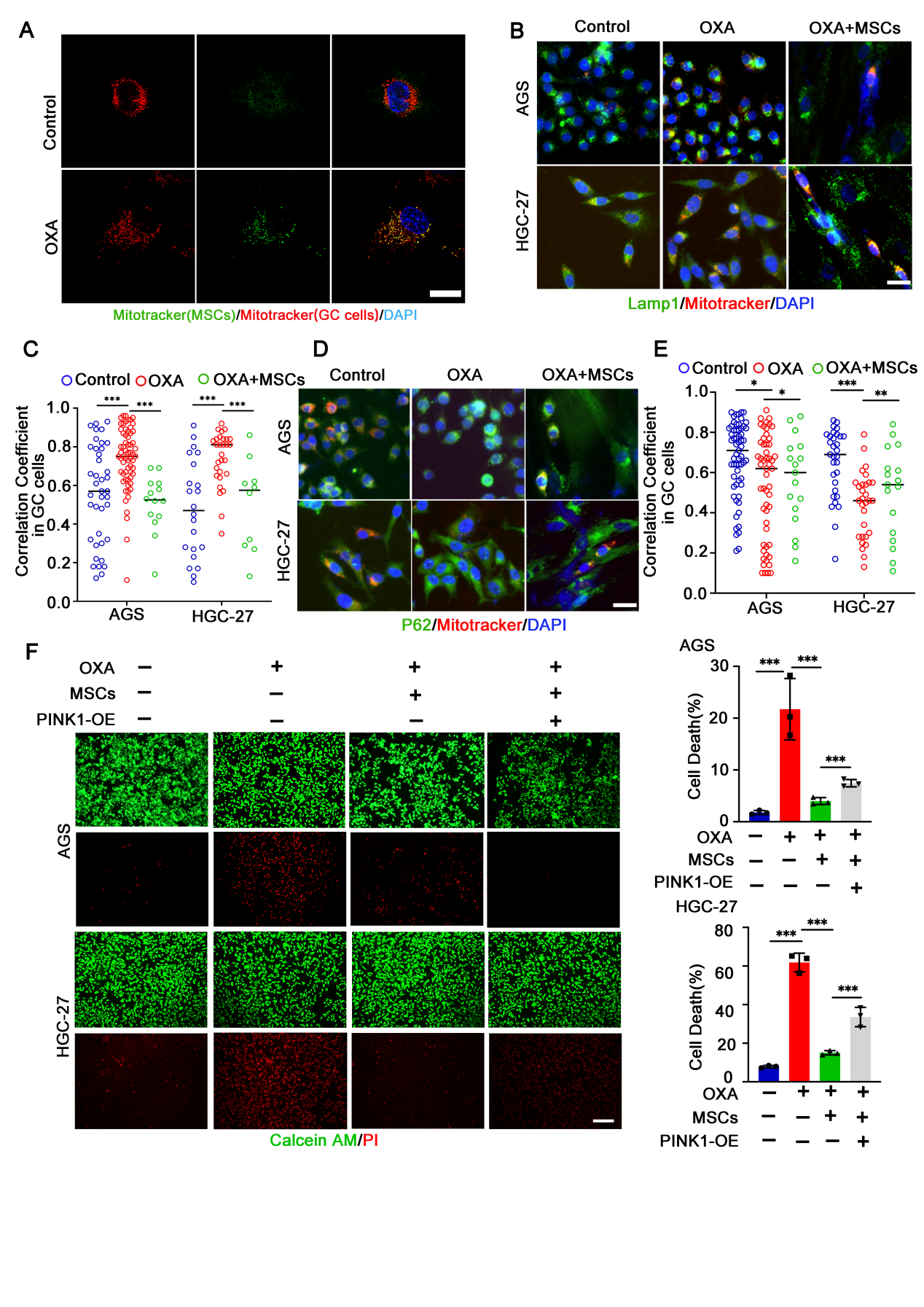


**Figure S5.** The cytotoxic effect of OXA on GC cells was weakened after tumor received mitochondrial transfer.

(A) Representative immunostaining images of fused mitochondria in each group (MSCs-AGS and MSCs-AGS+OXA) by Structure Illumination Microscopy (SIM) (n = 3-4). MitoTracker Red-labeled MSCs and MitoTracker Deep Red-labeled GCs. Scale bar: 20 µm. (B) Representative immunostaining images of Lamp1-stained GC cells in each group (GC cells, GC cells+OXA, and MSCs-GC cells+OXA). Scale bar: 20 µm. (C) Quantification of the levels of colocalization in (B) and correlation coefficient shown under each condition (n = 3-4). (D) Representative immunostaining images of P62-stained GC cells in each group (GC cells, GC cells+OXA, and MSCs-GC cells+OXA). Scale bar: 20 µm. (E) Quantification of the levels of colocalization in (D) and correlation coefficient shown under each condition (n = 3-7). (F) Representative images of calcein-AM/PI staining in each group (GC cells, GC cells+OXA, MSCs-GC cells+OXA, and MSCs-GC cells+OXA+PINK-OE) (n = 3). Scale bar: 100 µm. The data above are presented as mean ± S.D. of three independent experiments. P-values are calculated between two groups was performed using an unpaired *t*-test, and multiple-group statistical analysis was performed using one-way analysis of variance (anova) followed by the Tukey multiple-comparison test. ns, not significant; *P<0.05; **P<0.01; ***P<0.001.


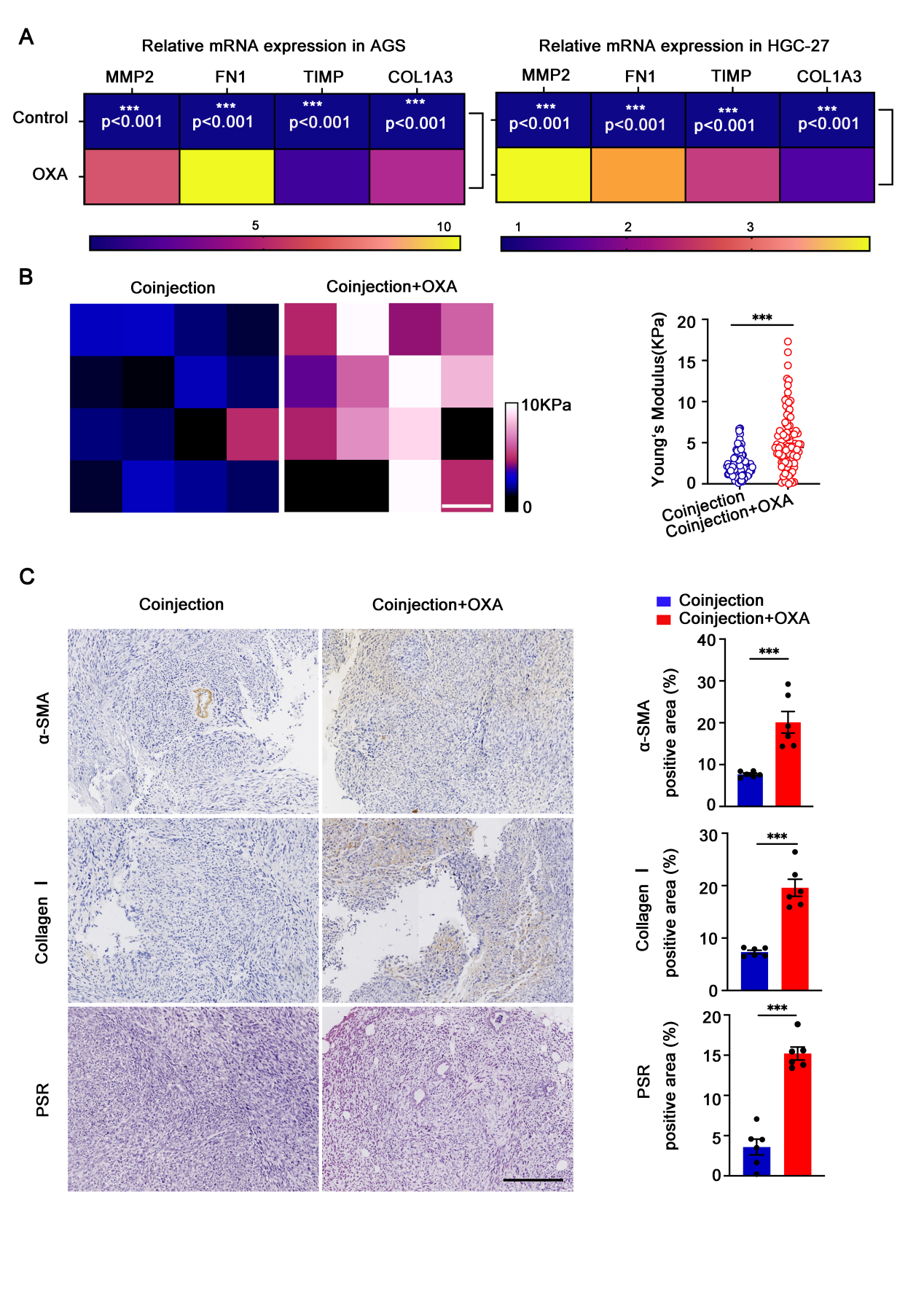


**Figure S6.** OXA treatment promoted the expression of ECM-related genes in GC cells and increased the matrix stiffness in mice.

(A) qRT-PCR analysis of MMP2, FN1, TIMP, and COL1A3 mRNA levels in GC cells (n = 3). (B) Atomic force images and statistical analysis *in vivo* (n = 6 mice/group). Scale bar: 1 µm. (C) Representative IHC images of GC tissues in mice, and quantitative analysis of levels of α-SMA, collagen Ⅰ and PSR. The images were obtained with a 4× magnification lens. Scale bar: 200 µm. (n = 6 mice/group). The data above are presented as mean ± S.D. of three independent experiments. *P*-values are calculated between two groups was performed using an unpaired *t*-test, and multiple-group statistical analysis was performed using one-way analysis of variance (anova) followed by the Tukey multiple-comparison test. ns, not significant; **P*<0.05; ***P*<0.01; ****P*<0.001.

**
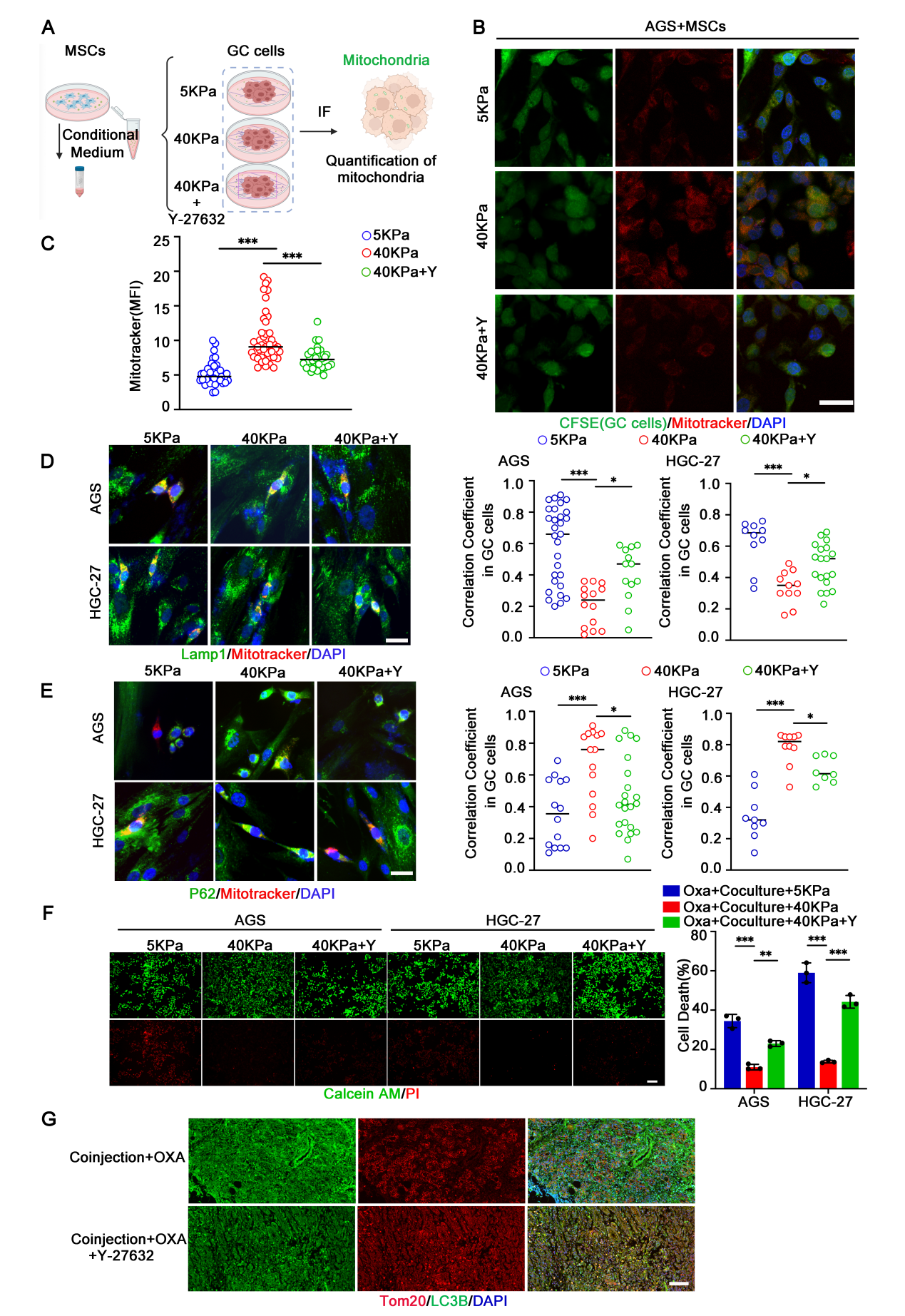
**

**Figure S7.** Targeting RhoA/ROCK1 signaling pathway restores mitophagy levels of GC cells on high matrix stiffness.

(A) Schematic overview of conditioned medium. (B-C) Representative immunofluorescence images. Scale bar: 30 μm. Quantitative analysis of the mitochondrial transfer in each group (5 KPa, 40KPa, 40KPa+Y27632) (n = 3). (D) Representative immunofluorescence images displayed the colocalization of mitochondria (Red) and Lamp1 (Green) under different conditions. Scale bar: 20 μm. Quantification of the levels of colocalization, and correlation coefficient shown on the right (n = 4-8). (E) Representative immunofluorescence images displayed the colocalization of mitochondria (Red) and P62 (Green) under different conditions. Scale bar: 20 μm. Quantification of the levels of colocalization, and correlation coefficient shown on the right (n = 3-4). (F) Representative images in each group using calcein-AM/PI staining, and the mortality rate of GC cells in each group after 24 hour treatment (n = 3). Scale bar: 100 μm (n = 3). (G) Representative immunostaining images of LC3B and Tom20 staining in each group (HGC-27-MSCs+OXA, HGC-27-MSCs+OXA+Y-27632) (n = 6 mice/group). Scale bar: 100 μm. The data above are presented as mean ± S.D. of three independent experiments. *P*-values are calculated between two groups was performed using an unpaired *t*-test, and multiple-group statistical analysis was performed using one-way analysis of variance (anova) followed by the Tukey multiple-comparison test. ns, not significant; **P*<0.05; ***P*<0.01; ****P*<0.001.

**Table S1.** Antibodies, Chemical reagents and Kits

| **Antibodies** | **Company** | **Cat. No** |
| --- | --- | --- |
| PGC-1α | Abcam | ab191838 |
| TFAM | Abcam | ab176558 |
| TOM20 | Santa Cruz | sc-17764 |
| Lamp1 | Santa Cruz | sc-19992 |
| P62 | Abcam | ab56416 |
| α-SMA | CST | 19245S |
| Collagen I | CST | 72026S |
| Annexin-A1 | CST | 32934 |
| COX4 | Santa Cruz | sc-376731 |
| Tom20 | Santa Cruz | sc-17764 |
| Goat Anti-Rabbit IgG H&L (HRP) | Abcam | ab205718 |
| Goat Anti-Mouse IgG H&L (HRP) | Abcam | ab205719 |
| Alpha Actin Polyclonal antibody | proteintech | 23660-1-AP |
| **Chemical reagents** |  |  |
| Annexin-V-APC | Biolegend | 640919 |
| Propidium Iodide Solution | Biolegend | 421301 |
| Alexa Fluor™ 488 Phalloidin | Thermo | A12379 |
| MitoSox Red | Invitrogen | M36008 |
| DAPI Solution | Sigma | D9542-1mg |
| Annexin V Binding Buffer | Beyotime | 422201 |
| TRIzol | Thermo | 15596018 |
| PMSF Solution (100mM) | Beyotime | ST507-10ml |
| Oxaliplatin | MCE | HY-17371 |
| DAB | MXB | DAB-1031 |
| Mitotracker Deep Red | Invitrogen | M22426 |
| Mitotracker Red | Invitrogen | M22425 |
| **Kits** |  |  |
| Cell Mitochondria Isolation Kit | Beyotime | C3601 |
| Enhanced ATP Assay Kit | Beyotime | S0027 |
| Picro Sirius Red Stain Kit | MKBio | MM004-100ML |
| CellTrace™ CFSE Cell Proliferation Kit | Thermo | C34570 |
| Calcein/PI Cell Viability/Cytotoxicity Assay Kit | Beyotime | C2015S |
| Mitochondrial Membrane Potential Assay Kit with TMRE | Beyotime | C2001S |
| TUNEL assay Kit | Beyotime | C1086 |

**Table S2.** Primers used for real-time PCR

| TFAM-Forward | CTGTCTAAGAACTGGTCCGATG |
| --- | --- |
| TFAM-Reverse | TACTTTGTTCGCTCCTCCAC |
| PPARGC1A-Forward | TCCTCTGACCCCAGACTCAC |
| PPARGC1A-Reverse | TAGAGTCTTGGAGCTCCT |
| NRF1-Forward | TTGGAGAATGTGGTGCGTAAGT |
| NRF1-Reverse | GAGAGGCGGCAGTTCTGAGT |
| COL1A3-Forward | TTGAAGGAGGATGTTCCCATCT |
| COL1A3-Reverse | ACAGACACATATTTGGCATGGTT |
| FN1-Forward | GATGTCCGA ACAGCTATTTACCA |
| FN1-Reverse | CGACCACATAGGAAGTCCCAG |
| MMP2-Forward | CCTCCCGGTGCCCAAGAATAGA |
| MMP2-Reverse | GGCTCTGAGGGTTGGTGGGATT |
| TIMP1-Forward | TGGAAAACTGCAGGATGGACTCTTG |
| TIMP1-Reverse | CAGGGGATGGATAAACAGGGAAACA |
